# Direct coupling of oligomerization and oligomerization-driven endocytosis of the dopamine transporter to its conformational mechanics and activity

**DOI:** 10.1016/j.jbc.2021.100430

**Published:** 2021-02-18

**Authors:** Tatiana Sorkina, Mary Hongying Cheng, Tarique R. Bagalkot, Callen Wallace, Simon C. Watkins, Ivet Bahar, Alexander Sorkin

**Affiliations:** 1 Department of Cell Biology, School of Medicine, University of Pittsburgh, Pittsburgh, Pennsylvania, USA; 2 Department of Computational and Systems Biology, School of Medicine, University of Pittsburgh, Pittsburgh, Pennsylvania, USA

**Keywords:** dopamine transporter, oligomerization, endocytosis, molecular modeling, AL, AIM-100–like, BS^3^, bis(sulfosuccinimidyl)suberate, DA, dopamine, DAT, dopamine transporter, DMSO, dimethyl sulfoxide, EC, extracellular, EGFR-GFP, GFP-tagged epidermal growth factor receptor, FRET, fluorescence resonance energy transfer, FRETN, normalized sensitized fluorescence resonance energy transfer, GNM, Gaussian network model, HBSS, Hanks balanced salt solution, IC, intracellular, IF, inward-facing, KRHG, glucose-supplemented Krebs-Ring buffer, MD, molecular dynamics, NET, norepinephrine transporter, NSS, neurotransmitter sodium symporter, OF, outward-facing, OFc, OF *closed*, OF*o*, outward-facing open, PAE, porcine aortic endothelial, ROI, regions of interest, SERT, serotonin transporter, TM, transmembrane, YFP-HA-DAT, YFP- and HA-epitope tagged DAT

## Abstract

Dopamine transporter (DAT) mediates the reuptake of synaptically released dopamine, and thus controls the duration and intensity of dopamine neurotransmission. Mammalian DAT has been observed to form oligomers, although the mechanisms of oligomerization and its role in DAT activity and trafficking remain largely unknown. We discovered a series of small molecule compounds that stabilize trimers and induce high-order oligomers of DAT and concomitantly promote its clathrin-independent endocytosis. Using a combination of chemical cross-linking, fluorescence resonance energy transfer microscopy, antibody-uptake endocytosis assay, live-cell lattice light sheet microscopy, ligand binding and substrate transport kinetics analyses, and molecular modeling and simulations, we investigated molecular basis of DAT oligomerization and endocytosis induced by these compounds. Our study showed that small molecule–induced DAT oligomerization and endocytosis are favored by the inward-facing DAT conformation and involve interactions of four hydrophobic residues at the interface between transmembrane (TM) helices TM4 and TM9. Surprisingly, a corresponding quadruple DAT mutant displays altered dopamine transport kinetics and increased cocaine-analog binding. The latter is shown to originate from an increased preference for outward-facing conformation and inward-to-outward transition. Taken together, our results demonstrate a direct coupling between conformational dynamics of DAT, functional activity of the transporter, and its oligomerization leading to endocytosis. The high specificity of such coupling for DAT makes the TM4-9 hub a new target for pharmacological modulation of DAT activity and subcellular localization.

The plasma membrane dopamine transporter (DAT) regulates dopamine (DA) neurotransmission in the central nervous system by mediating the reuptake of DA following its release by dopaminergic neurons (1, 2). Thus, DAT plays a critical role in many functions of the brain DA system including locomotion control, motivation, and reward-seeking behaviors (3, 4). DAT is a primary target for psychostimulants, *e.g.,* cocaine and amphetamines, and is involved in neurological disorders and neurodegenerative pathologies (5, 6, 7, 8).

DAT and other monoamine transporters belong to the solute carrier 6 (SLC6) family of Na^+^-dependent neurotransmitter symporters (9). They are composed of 12 transmembrane (TM) helices with both amino termini and carboxy termini projected intracellularly (10, 11, 12). The molecular mechanism of substrate transport by neurotransmitter sodium symporter (NSS) family members has been extensively studied using biochemical, structural, and computational approaches. Our current understanding is an alternating access mechanism where binding of the substrate and Na^+^ ions to the extracellular (EC) vestibule of DAT in the outward-facing (OF) state triggers the transition to an occluded intermediate followed by an inward-facing (IF) state. The IF state, in turn, allows for the release of substrate and cations to the intracellular (IC) medium; and the transport cycle is completed by the return of DAT to its OF state (13, 14).

Despite significant progress in characterizing DAT structure and function (13, 14), many aspects of DAT regulation remain to be understood. Perhaps one of the most significant limitations in our understanding is the process of DAT oligomerization and its role in DAT function and localization in the cell. For example while *Drosophila* DAT was crystallized as a monomer (12), biochemical and microscopy studies provide evidence for dimerization, high-order oligomerization, and cluster assembly of mammalian DAT expressed in nonneuronal cells and endogenous rodent DAT in dopaminergic neurons (15, 16, 17, 18, 19, 20, 21, 22, 23, 24, 25, 26). Furthermore, the molecular mechanisms mediating and regulating DAT oligomerization and their relation to substrate transport properties and IC trafficking remain largely unknown.

We recently showed that furopyrimidine AIM-100, an inhibitor of the cytoplasmic activated CDC42 tyrosine kinase (ACK1/*TNK2*), induced dramatic oligomerization and clustering in cellular membranes of human DAT stably expressed in various cultured cells and endogenous mouse DAT (25). It was proposed that oligomerization and clustering promoted DAT clathrin-independent and dynamin-independent endocytosis, which did not require ACK1 activity. However, ACK1 has been proposed to regulate constitutive DAT endocytosis (27, 28). ACK1 has also been implicated in the regulation of endosomal sorting of signaling receptors (29). Therefore, the involvement of ACK1, if any, remained unclear as it might be conceivable that AIM-100–induced accumulation of DAT in endosomes resulted from a combination of ACK1-dependent and ACK1-independent effects.

To eliminate the confounding effect of ACK1 and analyze ACK1-independent mechanisms that underlie DAT endocytosis, we searched for AIM-100–like (AL) compounds that induce DAT oligomerization and endocytosis but do not inhibit ACK1. Our analysis using these small molecules and DAT mutagenesis revealed the key role of the preferred conformational state and dynamics of DAT as well as selected amino acids in TM helices TM4 and TM9 in augmenting oligomerization and promoting endocytosis. The results expose a striking coupling between intramolecular conformational dynamics, intermolecular association, and endocytosis events and suggest novel strategies for modulating DAT activity.

## Results

### AIM-100–like small-molecules increase DAT oligomerization

**Figure 1:**
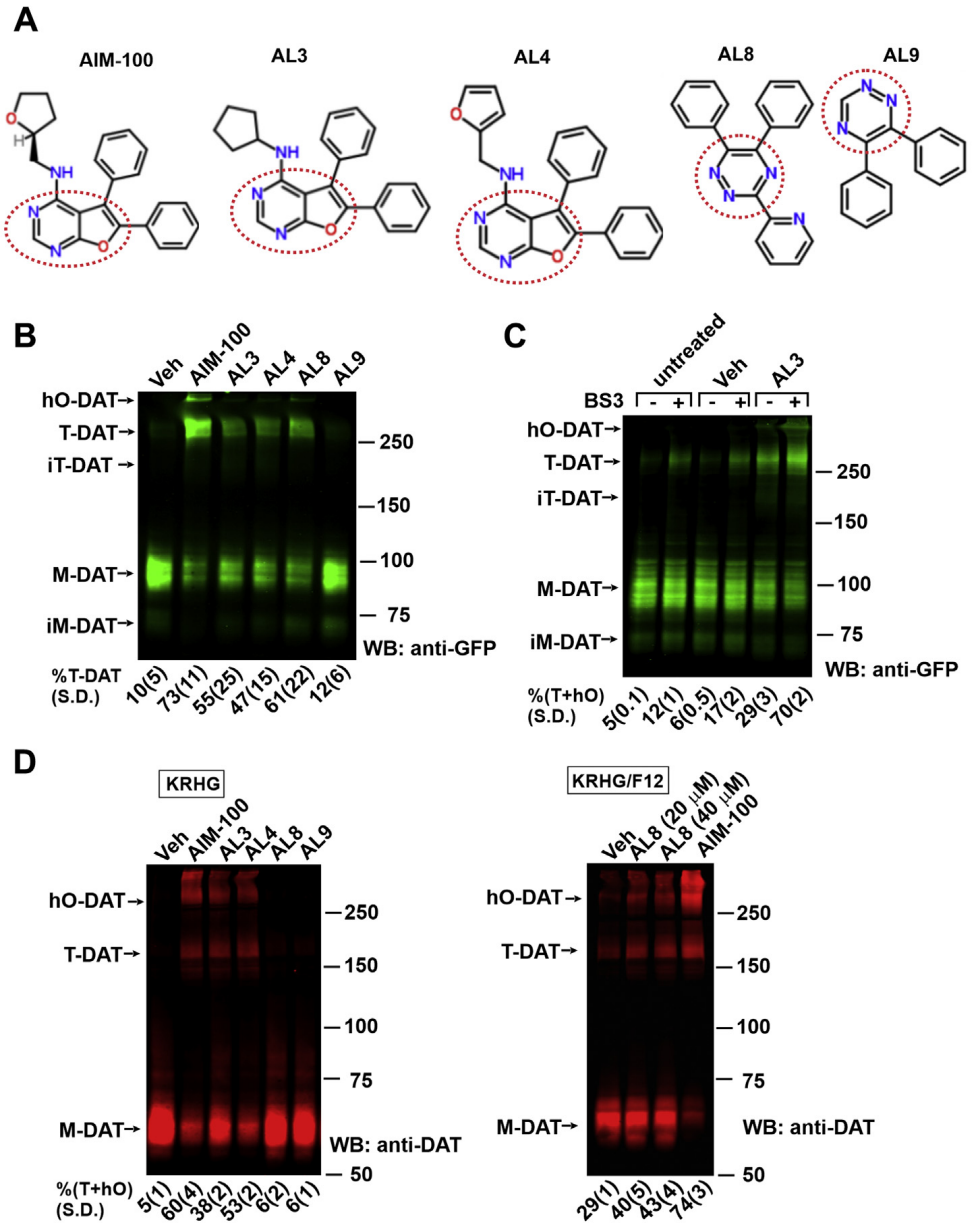
**ALs enhance DAT oligomerization**. *A,* structure of AIM-100, AL3, AL4, AL8, and AL9. Central scaffolds are indicated by *red* punctuate lines. See Fig. S1 for the full list, names, and structures of all tested compounds. *B,* PAE/YFP-HA-DAT cells were incubated with vehicle (*Veh*; DMSO), AIM-100, AL3, AL4, AL8, or AL9 (all 20 μM) at 37 °C for 2 h. Lysates were resolved by SDS-PAGE and probed by Western blotting with the GFP antibody. Mean values (±SD) of the fraction of trimers [T-DAT] of total mature YFP-HA-DAT (M-DAT+T-DAT) immunoreactivity are shown below the GFP blot (n = 3). *C,* PAE/YFP-HA-DAT cells were untreated or incubated with vehicle (*Veh*; DMSO) or AL3 (20 μM) at 37 °C for 1 h, washed with KRHG and incubated with 4 mM BS^3^ for 15 min at 37 °C in KRHG. Lysates were resolved by SDS-PAGE and probed by western blotting with the GFP antibody. Mean values (±SD) of the fraction of trimers [T+hO] of total mature YFP-HA-DAT immunoreactivity are shown below the GFP blot (n = 3). *D,* striatal synaptosomes prepared from HA-DAT mice were incubated with vehicle, AIM-100, AL3, AL4, AL9 (all 20 μM) or AL8 (20–40 μM) in KRHG (*left panel*) or KRHG/F12 (1:1) (*right panel*) at 37 °C for 2 h. Lysates were resolved by electrophoresis and probed by Western blotting with the DAT antibody. Mean values (±SD) of the fraction of trimers and high-order oligomers (T+hO) of total HA-DAT immunoreactivity were calculated from the data obtained in three mice that were treated in independent experiments (n = 3). AL, AIM-100–like; DAT, dopamine transporter; DMSO, dimethyl sulfoxide; h*O*, high order oligomers; *im-M*, immature monomers; *im-T*, immature trimers; KRHG, glucose-supplemented Krebs-Ring buffer; *M*, monomers; PAE, porcine aortic endothelial; *T*, trimers; YFP-HA-DAT, YFP- and HA-epitope tagged DAT.

Treatment of cells with AIM-100 induces DAT oligomerization, detected by SDS-PAGE as a robust accumulation of high molecular mass SDS-resistant species and promotes DAT clustering in the plasma membrane and endocytosis (25). To examine the ability of AL compounds, shortly designated as ALs, to induce DAT oligomerization and endocytosis, we performed screening assays with 16 small molecules in porcine aortic endothelial (PAE) cells stably expressing YFP- and HA-epitope tagged DAT (YFP-HA-DAT) (18, 30). These compounds were structurally similar to AIM-100 with or without the two phenyl groups attached to a central scaffold, with a variable third side chain or with di-phenyl-modified central scaffold, including the DA reuptake inhibitor, GBR12935 (Figs. 1*A* and S1). Among ALs, AL3, 4 and 8 caused the largest redistribution of the YFP-HA-DAT immunoreactivity—from monomeric ∼90 to 100 kDa (M-DAT) to ∼270 to 300 kDa and additional larger species (Fig. 1*B*), even though the extent of oligomerization was typically smaller than that induced by AIM-100. We will refer to the 270 to 300 kDa SDS-resistant species as a trimer (T-DAT), notwithstanding that it could be an oligomer of a different stoichiometry in intact cells. A small amount of the same trimer species was detected in lysates of vehicle-treated cells (Fig. 1*B*). Importantly, chemical cross-linking with bis(sulfosuccinimidyl)suberate (BS^3^) in untreated or vehicle-treated cells significantly increased the fraction of YFP-HA-DAT trimers (Fig. 1*C*). Likewise, BS^3^ amplified AL-induced increase in DAT trimerization and high-order oligomerization (example with AL3 is shown; Fig. 1*C*). Cross-linking did not result in the appearance of a band, corresponding to YFP-HA-DAT dimer, indicating that the 270 to 300 kDa species is a minimal BS^3^-cross-linkable constitutive oligomer that can be stabilized by ALs.

AL3 and AL4 are highly similar to AIM-100 with furopyrimidine as the central scaffold, whereas AL8 has a different scaffold—triazine (Fig. 1*A*). Triazine-based AL9 that lacks the pyridyl sidechain (Fig. 1*A*) was ineffective and used as the negative control to AL8 (Fig. 1*B*). The effects on DAT oligomerization were studied at concentrations of AL3, AL4, and AL8 of 10 to 40 μM. For consistency with previous work (25), we adopted concentrations of 20 to 40 μM for all ALs in subsequent experiments. However, AL3, AL4, and AL8 as well as AIM-100 are poorly soluble in aqueous liquids (see Fig. S1). As a result, they had effects of variable strengths on DAT and may be equally effective at lower concentrations.

AL3 and AL4 significantly increased oligomerization of endogenous HA-DAT in mouse striatal synaptosomes isolated from HA-DAT knock-in mice (31) (Fig. 1*D*; left panel). The minimal SDS-resistant oligomer was ∼200 kDa, which precisely corresponds to a trimer of the 65 kDa monomeric HA-DAT. AL8 did not induce significant DAT oligomerization in synaptosomes that were routinely prepared and maintained in the glucose-supplemented Krebs-Ring buffer (KRHG) (Fig. 1*D*; left panel) but increased the fraction of HA-DAT oligomers by 35% (SD = 5%) in the presence of 1:1 mixture of KRHG and F12 medium (Fig. 1*D*, right panel). Given the metal-binding properties of AL8 (32), it is possible that the presence of metals in F12 medium was necessary for its effect on DAT.

### ALs induce DAT endocytosis

**Figure 2:**
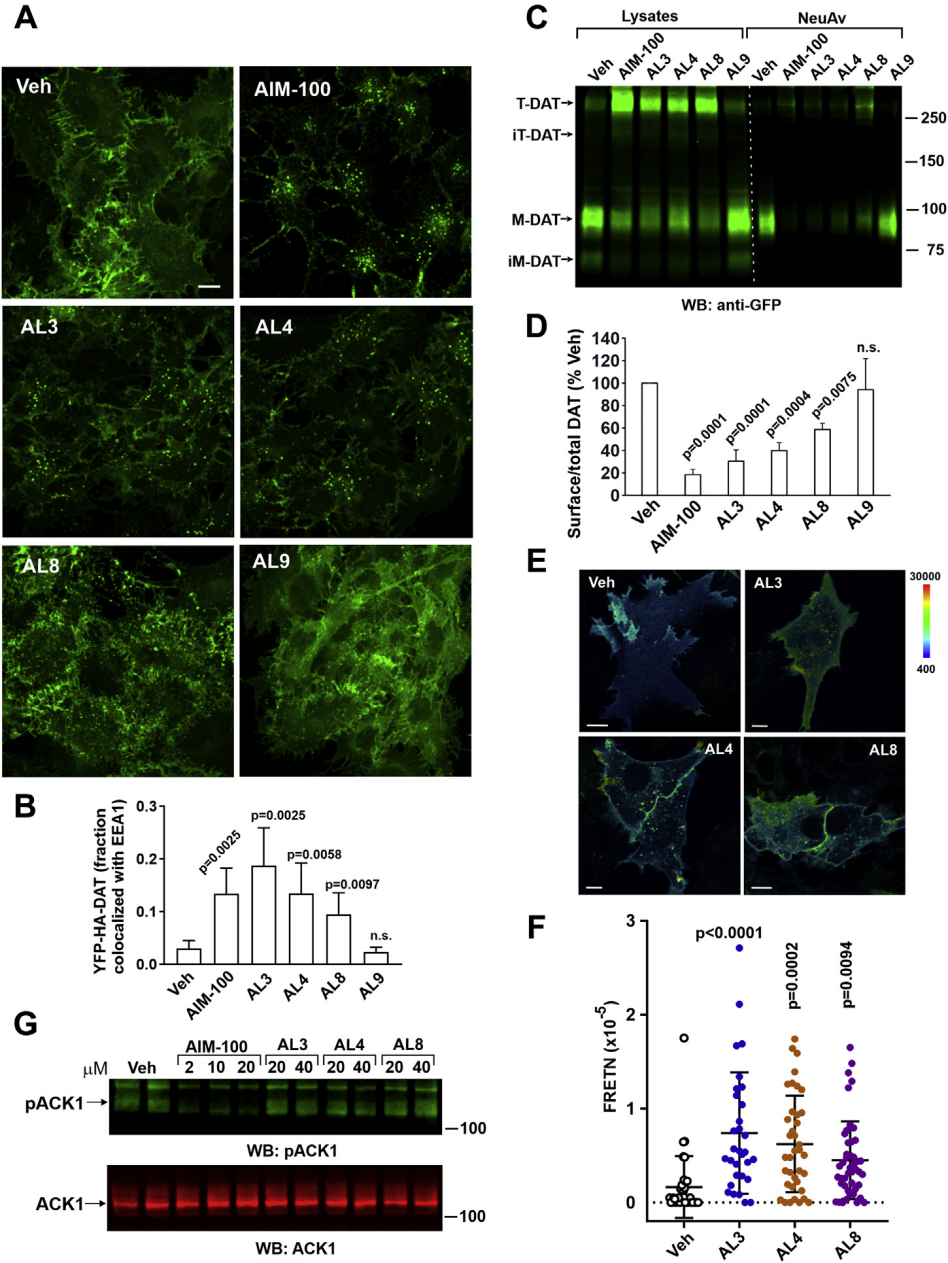
**ALs induce DAT endocytosis and accumulation of DAT oligomers in endosomes but do not inhibit ACK1**. *A*, PAE/YFP-HA-DAT cells were incubated with vehicle (DMSO), AIM-100, AL3, AL4, AL8, or AL9 (all 20 μM) for 2 h at 37 °C. 3D images were acquired from fixed cells through the 488-nm (YFP, *green*). Maximum intensity projections of 3D images are presented. *Scale bar*, 10 μm. Data are representative of five independent experiments. *B*, PAE/YFP-HA-DAT cells were incubated with vehicle (DMSO), AIM-100, AL3, AL4, AL8, or AL9 (all 30 μM) for 2 h at 37 °C, fixed and immunolabeled with EEA.1 antibody (EEA1). 3D images were acquired through the 488 nm (YFP, *green*) and 561 nm (EEA1, *red*) channels. Maximum intensity projections of 3D images are presented in Fig. S2. Fraction of total cellular YFP-HA-DAT co-localized with EEA1 was calculated as described in “Experimental Procedures”. Bar graph represents mean values with SDs (n of 6 images with multiple cells). The *p* values against vehicle treatment were calculated using multiple comparison one-way ANOVA. *C* and *D*, PAE/YFP-HA-DAT cells were incubated with DMSO (*Veh*), AIM-100 (20 μM), AL3 (40 μM), AL4 (40 μM), AL8 (40 μM), or AL9 (40 μM) for 2 h at 37 °C and biotinylated at 4 °C as described in “Experimental procedures”. Lysates were incubated with NeutroAvidin (*NeuAv*) to pull-down biotinylated proteins. Aliquots of lysates and NeuAv pulldowns were resolved by SDS-PAGE electrophoresis and probed by Western blotting with GFP antibodies. Representative Western blot image of three independent experiments is shown in (*C*). In (*D*), fractions of the biotinylated mature DAT (M-DAT plus T-DAT) relative to total mature DAT (*Lysates*) were quantified in 3 independent experiments and presented as percent of this fraction in “Veh” samples (mean ± S.D.). The *p* values against “*Veh”* were calculated using multiple comparison one-way ANOVA. *E* and *F*, PAE cells stably co-expressing CFP-DAT and YFP-DAT were incubated with DMSO (*Veh*), AL3, AL4, or AL8 (all 20 μM) for 90 min at 37 °C, and FRET imaging was performed at room temperature (20 °C). *E*, FRET^C^ images were calculated as described in “Experimental procedures” and are presented as pseudocolor intensity-modulated images (FRET^C^/CFP). The intensity scales of the FRET^C^ and CFP fluorescence are the same in all images. Scale bars, 10 μm. *F*, Graph represents FRETN values of individual cell regions, structures and endosomal compartments measured in vehicle- and AL-treated cells (±SD; n = 35–45). The *p* values were calculated against “*Ve*h” using one-way ANOVA with multiple comparisons. *G*, HeLa cells were stimulated with 100 ng/ml EGF for 10 min at 37 °C after preincubation with vehicle, AIM-100, AL3, AL4, or AL8. Lysates were resolved by SDS-PAGE and probed by Western blotting with antibodies to phosphoTyr284 ACK1 and total ACK1. Data are representative of three independent experiments. AL, AIM-100–like; DAT, dopamine transporter; DMSO, dimethyl sulfoxide; FRETN, normalized sensitized fluorescence resonance energy transfer; h*O*, high order oligomers; *im-M*, immature monomers; *im-T*, immature trimers; iM-DAT, immature monomers; iT-DAT, immature trimers; KRHG, glucose-supplemented Krebs-Ring buffer; *M*, monomers; M-DAT, mature monomers; *n.s.*, not significant; PAE, porcine aortic endothelial; *T*, trimers; T-DAT, mature trimers; YFP-HA-DAT, YFP- and HA-epitope tagged DAT.

Direct fluorescence microscopy imaging of YFP-HA-DAT showed that a 2-h treatment of cells with AL3 and AL4 strongly induced the endocytosis of YFP-HA-DAT (Fig. 2*A*). The accumulation of YFP-HA-DAT in vesicular compartments was accompanied by a reduction in its levels at the filopodium-like plasma membrane protrusions and ruffles (Fig. 2*A*). AL8 had a less pronounced effect on the appearance of YFP-containing vesicles, which were typically dimmer (apparent smaller size) as compared with those vesicles in cells treated with AIM-100 or AL3/4 (Fig. 2*A*). However, an accumulation of YFP-HA-DAT in vesicles induced by AL8 was evident when images were compared with those of cells treated with AL9 that had no visible effect on YFP-HA-DAT localization. Vesicular YFP-HA-DAT was significantly co-localized with early endosomal antigen EEA.1, a marker of early endosomes, in cells treated with AIM-100, AL3, AL4, or AL8 but not in cells treated with vehicle or AL9 (Figs. 2*B* and S2).

Cell-surface biotinylation assays confirmed robust AIM-100/AL-induced DAT endocytosis manifested by downregulation of the plasma membrane YFP-HA-DAT (Fig. 2, *C* and *D*). Notably, monomeric ∼70-kD (iM-DAT) and putative trimeric ∼200-kD (iT-DAT) species of immature YFP-HA-DAT were detected only in lysates but not in the plasma membrane fraction (“NeuAv”; Fig. 2*B*), indicating that both these species were of IC (endoplasmic reticulum) origin.

Prompted by observations of AL-induced oligomerization and endocytosis, we used fluorescence resonance energy transfer (FRET) microscopy to demonstrate the effect of ALs on DAT oligomerization in single living cells. Constitutive and AIM-100–induced oligomerization of DAT had been previously demonstrated by live-cell FRET microscopy (18, 25). We used the same method of sensitized FRET measurements in cells co-expressing CFP-DAT and YFP-DAT treated with ALs. The intensity of FRET from CFP to YFP was found to be significantly higher in the plasma membrane clusters and endosomes in AL3-, AL4-, and AL8-treated cells compared with that in vehicle-treated cells, where FRET measurements were taken in the regions of DAT accumulation, such as filopodia, ruffles, and cell–cell contacts. (Fig. 2, *E* and *F*). An increased FRET in AL-treated cells is indicative of the close proximity (<5 nm) of multiple DAT molecules, in support of AL-induced DAT oligomerization in living cells.

AL3, AL4, and AL8 did not cause any detectable SDS-resistant oligomerization and endocytosis of norepinephrine (NET) and serotonin transporters (SERT) (Fig. S3). This type of selectivity against DAT and not against other NSS family members is a feature shared with AIM-100 (25). Furthermore, to test whether ALs affect constitutive or stimuli-induced clathrin-mediated and clathrin-independent endocytosis of other cargoes, we used PAE cells stably expressing high level of GFP-tagged epidermal growth factor receptor (EGFR-GFP) (33). When stimulated with saturating ligand concentrations, EGFR has been shown to internalize via a clathrin-independent pathway (34). Fig. S4 demonstrates that ALs did not increase endocytosis of EGFR-GFP over its basal constitutive level and did not alter the clathrin-independent endocytosis of EGFR-GFP stimulated by 100 ng/ml EGF. In the same cells, clathrin-mediated endocytosis, assessed by imaging of the uptake of fluorescent transferrin, was also not affected by ALs (Fig. S4).

Importantly, unlike AIM-100, AL3, AL4, and AL8 did not inhibit the kinase activity of ACK1, as measured by probing the tyrosine autophosphorylation of ACK1 in HeLa cells in which ACK1 was activated upon cell stimulation with EGF (Fig. 2*G*). These data confirm that the effects of AIM-100 and ALs on DAT oligomerization and endocytic trafficking are independent of ACK1 activity.

### The ability of ALs to promote oligomerization and endocytosis is inhibited by cocaine

**Figure 3:**
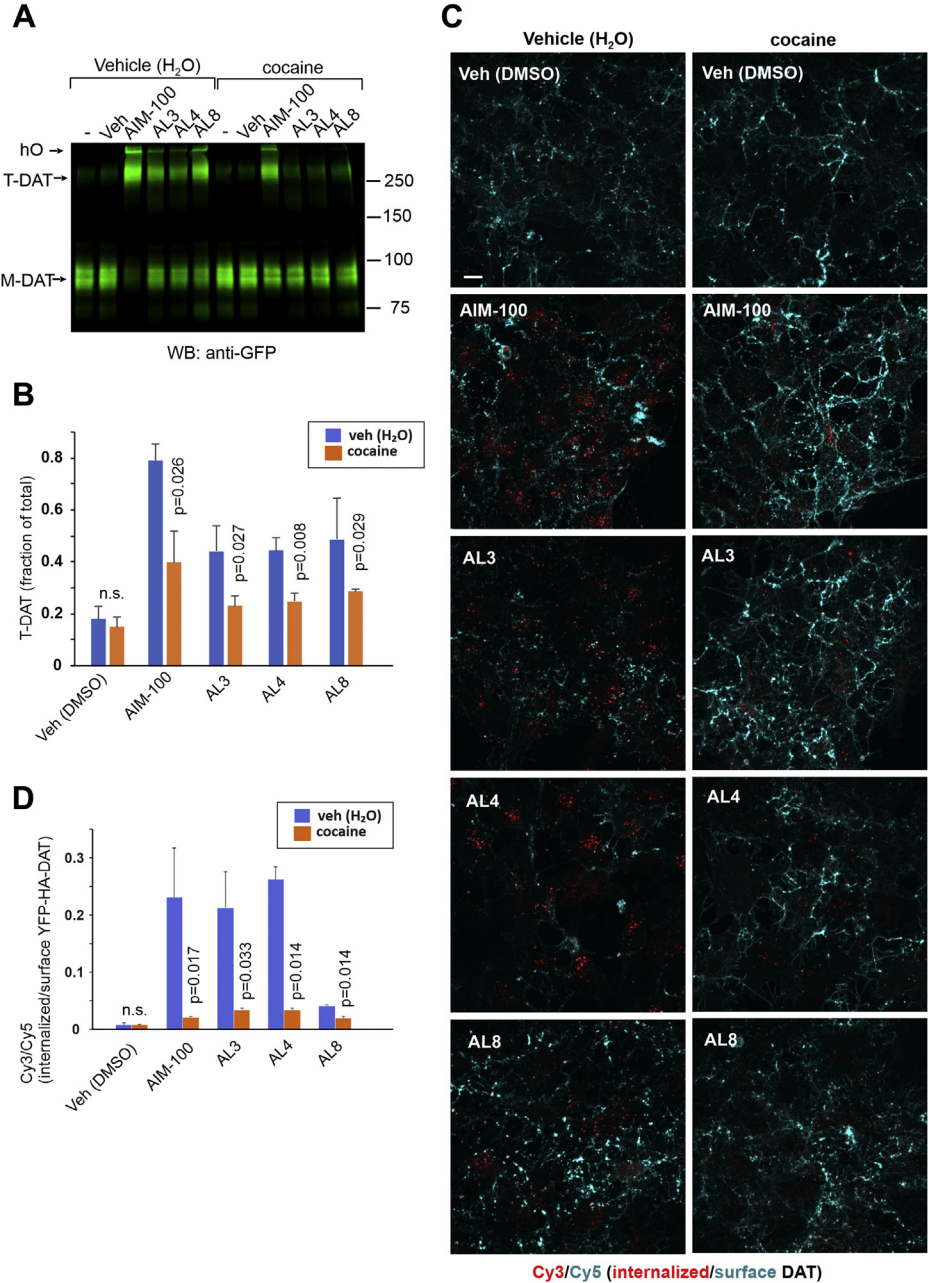
**Cocaine inhibits the oligomerization and endocytosis of DAT induced by ALs.***A*, PAE/YFP-HA-DAT cells were incubated with vehicle (water) or 20 μM cocaine for 10 min at 37 °C, and further incubated in the absence or presence of vehicle (DMSO), AIM-100, AL3, AL4, or AL8 (all 20 μM) with vehicle (water) (if not preincubated with cocaine) or 2 μM cocaine (preincubated) for 2 h at 37 °C, and lysates were analyzed by SDS-PAGE and immunoblotting with the GFP antibody. Representative experiment is shown. *B*, quantification of the fraction of ∼300 kDa trimeric species relative to the total mature YFP-HA-DAT. The mean values of the T-DAT fraction relative to total DAT (±SD; n = 3–4) were calculated from three independent experiments. The *p* values for “cocaine” compared with “vehicle (water)” variants were calculated using two-tail, paired *t* test. *C*, PAE/YFP-HA-DAT cells were incubated with HA11 for 30 min at 37 °C and then incubated as in (*A*). After fixation, cultures were stained with secondary Cy5-conjugated anti-mouse antibodies (surface HA-DAT), permeabilized with Triton X-100, and stained with secondary Cy3-conjugated anti-mouse antibody (internalized HA-DAT). 3D images were acquired through 488 (YFP, not shown), 561 (Cy3, *red*), and 640 nm (Cy5, *cyan*) channels. Maximum intensity projections of 3D images are presented. Scale bar, 10 μm. *D*, Cy3/Cy5 ratios were calculated in experiments exemplified in (*C*). Results are presented as mean values of the ratio (±SD; n = 3–4). The *p* values for “cocaine” compared with “vehicle (water)” variants were calculated using two-tail, paired *t* test. AL, AIM-100–like; DAT, dopamine transporter; DMSO, dimethyl sulfoxide; *M*, monomers; *n.s.*, not significant; PAE, porcine aortic endothelial; *T*, trimers; YFP-HA-DAT, YFP- and HA-epitope tagged DAT.

AIM-100–induced DAT oligomerization and endocytosis were shown to be inhibited by the DAT antagonist, cocaine (25). Here, we show that all effects of AL3, AL4, and AL8 on DAT were also inhibited by cocaine (Fig. 3). The inhibition of DAT oligomerization by cocaine was partial but significant in all experiments (Fig. 3, *A* and *B*). Cocaine strongly blocked small molecule–induced endocytosis measured using the HA antibody uptake assay (Fig. 3, *C* and *D*). Notably, the accumulation of YFP-HA-DAT in endosomes induced by 20 μM AL8 was statistically significant but much smaller than that in cells treated with AL3 or AL4 (Fig. 3*D*), although further increase in DAT endocytosis was observed at high AL8 concentrations (40 μM). Because AL8 had a weaker effect on DAT endocytosis, we classify it as “partially effective” (Fig. S1).

Altogether, the data in Figure 1, Figure 2, Figure 3 and their supplements demonstrate the DAT-specific and cocaine-sensitive effects of ALs on the oligomerization and endocytosis of recombinant human YFP-HA-DAT and endogenous mouse HA-DAT and confirm that these effects are not associated with the inhibition ACK1.

### TM4 and TM9 play key roles in mediating AL-induced oligomerization and endocytosis of DAT

**Figure 4:**
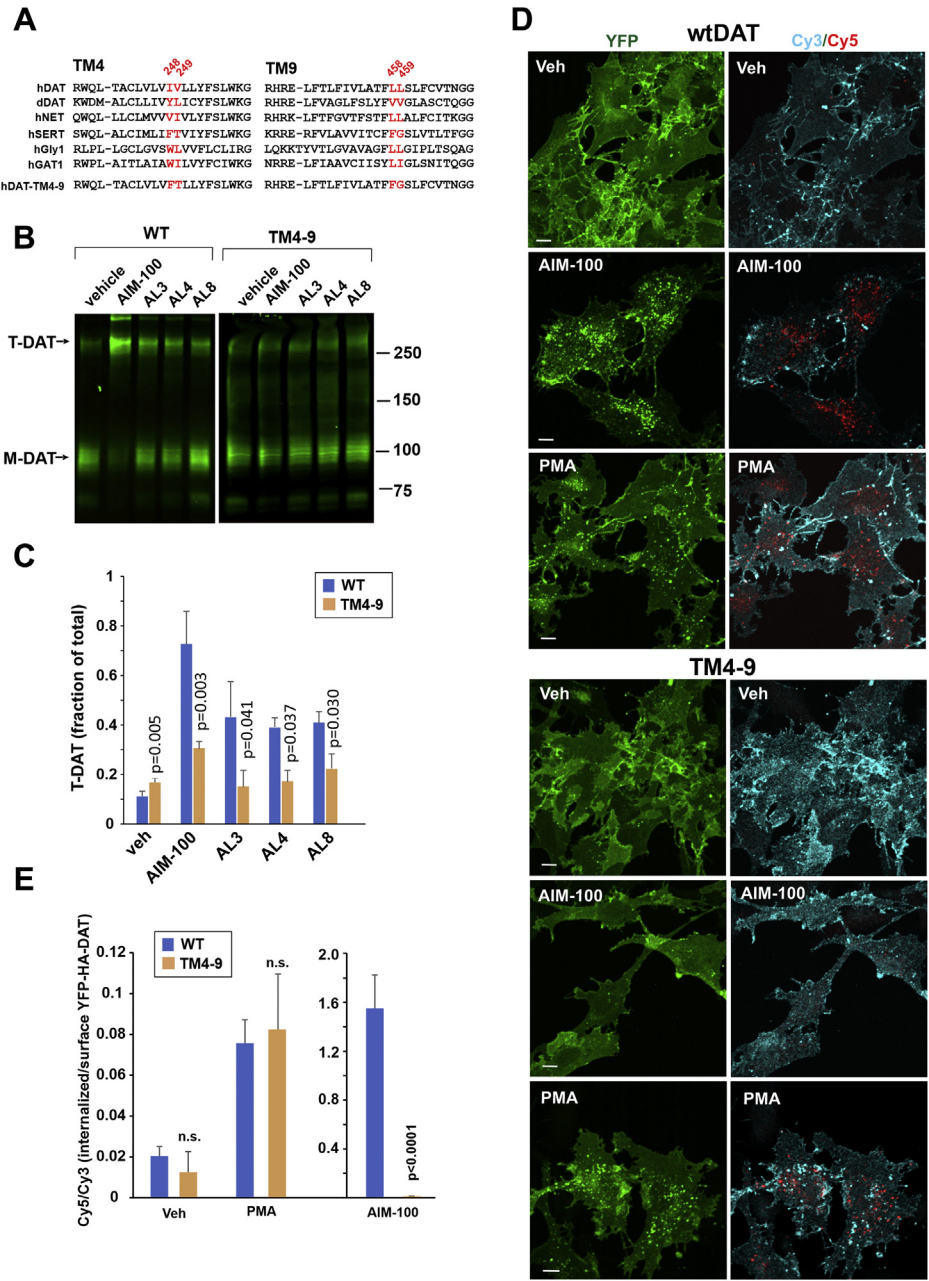
**Mutations in TM4 and TM9 inhibit the effects of AIM-100 and ALs on DAT.***A*, alignment of the TM4 and TM9 sequences of several SLC6 transporters and the TM4-9 mutant of human DAT. *B*, PAE/YFP-HA-DAT and PAE/TM4-9 cells were incubated with vehicle or AIM-100, AL3, AL4, or AL8 (all 20 μM) for 2 h at 37 °C, and lysates were analyzed by SDS-PAGE and immunoblotting with the GFP antibody. Representative experiment is shown. Blots of WT YFP-HA-DAT and TM4-9 are from the same gel. *C*, quantification of the fraction of trimeric species (*T*) of the total YFP-HA-DAT or TM4-9 DAT immunoreactivities. The mean values of the T fraction of total (±SD) were measured in four independent experiments. The *p* values were calculated for TM4-9 versus wtDAT using two-tail, paired *t* test. *D*, PAE/YFP-HA-DAT and PAE/TM4-9 cells were incubated with HA11 for 60 min at 37 °C and then incubated with vehicle (DMSO), 20 μM AIM-100 for 2 h or 1 μM PMA for 30 min, all at 37 °C. After fixation, cultures were stained with secondary anti-mouse antibodies conjugated with Cy3 (*surface HA-DAT*), permeabilized with Triton X-100, and stained with secondary anti-mouse conjugated with Cy5 (*internalized HA-DAT*). 3D images were acquired through 488 (YFP, *green*), 561 (Cy3, *cyan*), and 640 nm (Cy5, *red*) channels. Maximum intensity projections of representative 3D images are shown. Scale bars, 10 μm. *E*, Cy5/Cy3 ratios were calculated in images exemplified in (*E*). Results are presented as mean values of the ratio (±S.D.; n = 4). The *p* values were calculated for TM4-9 versus wtDAT using two-tail, paired *t* test. n.s., difference is not significant (*p* > 0.05). AL, AIM-100–like; DAT, dopamine transporter; DMSO, dimethyl sulfoxide; *im-M*, immature monomers; *im-T*, immature trimers; *M*, monomers; PAE, porcine aortic endothelial; PMA, phorbol 12-myristate 13-acetate; *T*, trimers; TM, transmembrane; YFP-HA-DAT, YFP- and HA-epitope tagged DAT.

To determine which DAT residues play a dominant role in the AIM-100/AL-induced oligomerization and endocytosis, we conducted site-directed mutagenesis experiments. Our recent study pointed to the involvement of TM4 and TM9, the latter containing a putative leucine heptad or “zipper” motif, in the AIM-100–induced stabilization of DAT trimer (26). Alanine-substitution of multiple TM9 leucine residues revealed that the L459 A mutation impaired AIM-100/AL-induced endocytosis when the mutant was transiently expressed. However, this effect was not statistically significant in cells stably expressing this mutant. Recent studies by Navratna *et al.* revealed that the mutation of I248 (TM4) to an aromatic residue resulted in a π-stacking interaction between F/Y248 and F457 of TM9 (35). The TM4–TM9 association prompted us to test the effects of the I248Y substitution. The I248Y mutant of YFP-HA-DAT displayed a partially reduced response to AIM-100 and ALs, although this reduction was again not statistically significant. Encouraged by the trends observed with I248Y and L459A mutants and because the AIM-100/AL-effects are unique to DAT, we generated a quadruple mutant where we replaced four TM4 and TM9 residues by their SERT counterparts: I248F, V249T, L458F, and L459G. This mutant will be shortly referred to as the “TM4-9” mutant (Fig. 4*A*). We hypothesized that the introduction of SERT-like residues, including two phenylalanines, into TM4 and TM9 of DAT could rewire the interactions between these helices, such that AIM-100/AL binding and/or AIM-100/AL-induced DAT oligomerization might be impeded.

In support of this hypothesis, we observed a substantial decrease in the fraction of oligomers (T-DAT) induced by AIM-100 and ALs in the TM4-9 mutant compared with that in the WT YFP-HA-DAT (Fig. 4, *B* and *C*). Moreover, total internal reflection fluorescence microscopy demonstrated only a minimal, if any, AIM-100 induced clustering of the TM4-9 mutant at the cell surface (Fig. S5). Interestingly, the constitutive oligomerization of the TM4-9 mutant (∼270–300 kDa species) was slightly increased compared with WT YFP-HA-DAT (Fig. 4, *B* and *C*).

In parallel with the decrease in AIM-100/AL-induced oligomerization, AIM-100 and ALs did not induce significant endocytosis of the TM4-9 mutant as observed on the images of direct YFP fluorescence and using the antibody uptake internalization assay (Figs. 4, *D*, *E* and S6). By contrast, endocytosis triggered by phorbol ester (phorbol 12-myristate 13-acetate) was similar in WT YFP-HA-DAT and TM4-9 mutant expressing cells (Fig. 4, *D* and *E*), demonstrating that this mutant was otherwise capable of clathrin-mediated endocytosis (36). Overall, the data in Figure 4 and its supplements showed that residues I248/V249 in TM4 and L458/L459 in TM9 are involved in the oligomerization and clathrin-independent endocytosis of DAT induced by AIM-100 and ALs.

### TM4-9 mutations alter the equilibrium conformations and dynamics of the transporter in favor of outward-facing state, prone to bind cocaine

The above experiments showed that cocaine binding and quadruple mutations had similar effects, both suppressing the oligomerization and internalization of DAT, which would be otherwise induced by AIM-100 or ALs. This observation suggests that both had a comparable effect on the molecular structure and mechanics, resulting in similar observables. To understand the mechanistic basis of the observed phenomena at the molecular level, we examined the change in the conformational dynamics of the quadruple mutant compared with that of WT DAT by carrying out a series of molecular dynamics (MD) simulations, in the presence of DA and co-transported ions (two Na+ and one Cl-).

**Figure 5:**
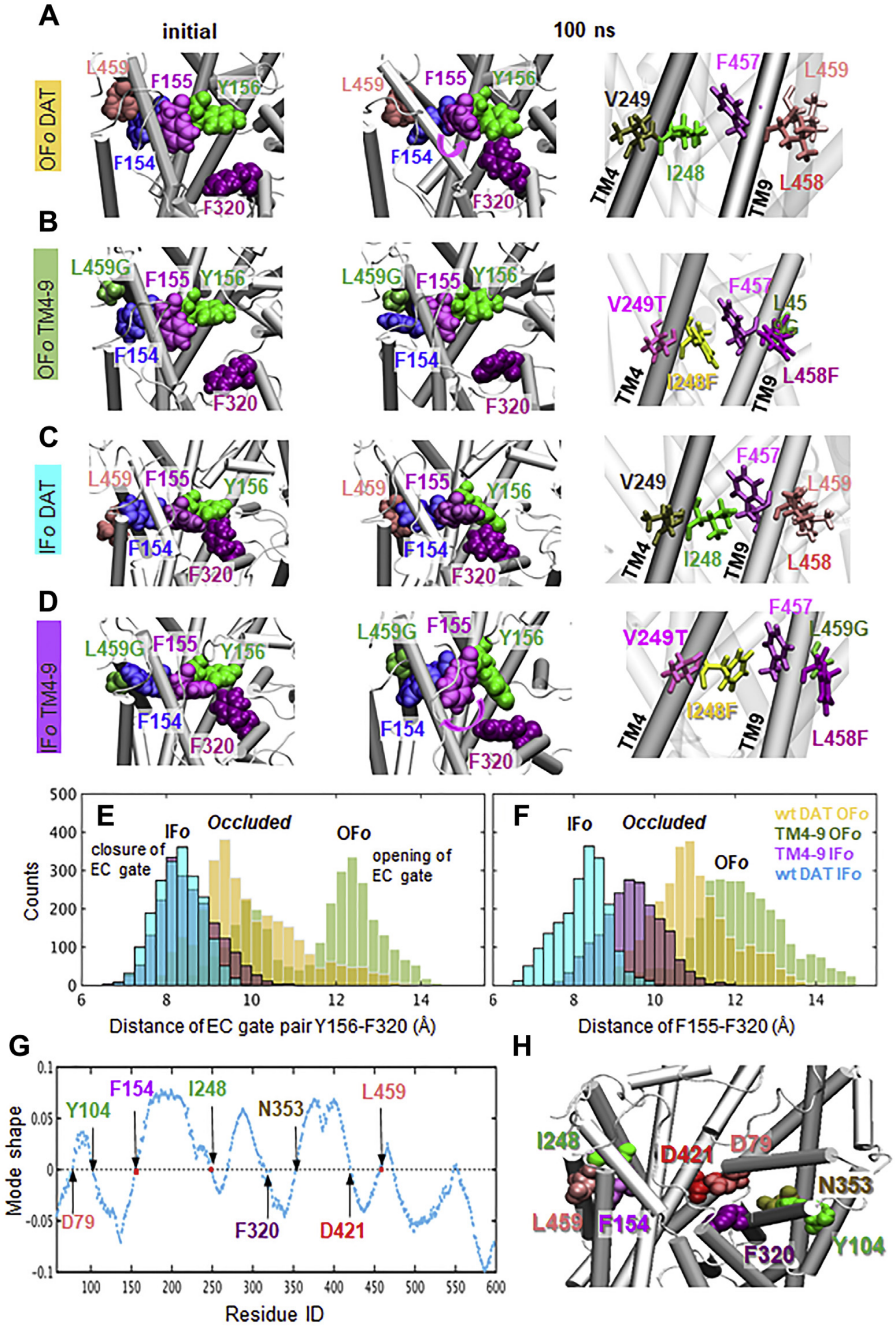
**TM4-9 mutations alter the conformational dynamics of the transporter in favor of the IF → occluded → OF transition**. Snapshots at t = 0 (*left*) and 100 ns (*middle*: viewed from EC region; *right:* side view) observed in MD simulations of (*A*) outward-facing open (OF*o*) wt DAT, (*B*) OF*o* TM4-9, (*C*) inward-facing open (IF*o*) wt DAT, and IF*o* (*D*) TM4-9 mutant. In the simulations of substrate/ion binding by the wt DAT in the OF state (*A*), the hydrophobic interaction between L459 and F154 facilitates the rotational isomerization of F155 in favor of the closure of the EC gate Y156-F320, thus promoting the progress to the occluded state. The mutation I248F in TM4-9 mutant leads to π-stacking interactions between I248F and F457, which stabilize the OF state (*B*; *right*), and the EC gate F320-Y156 remains open (*B*; *middle*). In the IF state (*C–D*), the hydrophobic interaction between L459 and F154 which stabilizes the WT DAT IF state is weakened in the mutant (where L459 is replaced by Gly). There is an increased flexibility at this region, and F155 undergoes a rotational isomerization such that the inner gate-forming residues Y156 and F320 open; the mutation thus facilitates the opening of the EC gate or the transition from IF toward the OF state. Cumulative histograms of the center of mass (COM) distances between (*E*) inner EC gate residues Y156 and F320; and (*F*) F155 and F320 observed in the simulations of the wt DAT OF (*orange with white line*), TM4-9 OF (*lime with white line*), wt DAT IF (*cyan with black line*) and TM4-9 IF (*purple with black line*), based on three runs for each OF conformer and two for IF conformer, each of duration 200 ns. In both cases, the mutant exhibits a transition towards larger values indicated by the *colored arrows*. *G*, The shape of GNM mode 1. The residues with values close to zero refer to structural regions that serve as hinges in the global mechanics of the transporter. Note that F154-F155, I248-V249, and L458-L459 serve as hinges (shown in *red squares*). Calculations in *G* were performed using *ProDy*. *H*, The residues participating in the global hinge, identified in *G*, displayed in vDW format, based on DAT OF model. DAT, dopamine transporter; GNM, Gaussian network model; IF, inward-facing; OF, outward-facing; TM, transmembrane.

Our earlier MD simulations (37) showed that binding of DA and ions to DAT triggers the closure of the EC gates (R85-D476 and Y156-F320) within 60 ± 30 ns, from OF *open* (OFo) to OF *closed* (OFc) state. Here OF refers to the overall architecture, and open/closed refer to the local changes in gate conformation. Gate closure and accompanying tight packing allosterically drive the transition of the OF transporter to an occluded (to both EC and IC environments) intermediate, followed by the IF*o* state. This behavior was consistently observed in three independent runs. In contrast, in the case of TM4-9 DAT mutant, the inner EC gate Y156-F320 remained open in multiple 100 ns runs (Fig. 5, *A, B* and *E*), whereas the outer EC gate R85-D476 (not shown) closed intermittently. Furthermore, the mutation I248F promoted π-stacking interactions between F248 and F457, which stabilize the OF state of the DAT monomer (35).

Further MD simulations starting from the IF state showed that TM4-9 mutations promote the transition from IF back to OF state (preventing full closure of the EC region). Notably, in both runs of IF*o* TM4-9 mutant, F155 sidechain underwent a rotational isomerization, from χ_1_ = 170 ± 15° (IF) to 60 ± 20° (OF), which enabled its dissociation from F320 (Fig. 5*D*); nevertheless, F155 remained in its IF orientation during the entire simulations of IF*o* WT DAT. Statistical examination of EC-gating pair Y156-F320 center of mass (COM) distances sampled during the simulations consistently demonstrated the strong tendency of the mutant to stabilize an OF open form (Fig. 5*E*). The same trend could be observed in histograms of the F155-F320 distances (Fig. 5*F*).

We consistently observed distinct local sidechain orientations of those mutated residues in different conformers (OF and IF), nevertheless the current runs of 200 ns each were too short to visualize the complete transport cycle of DAT. Therefore, we complemented MD simulations using Gaussian network model (GNM) method (38) to further interpret mutation effects. While GNM-based approaches lack atomic details, they provide robust analytical solutions for the collective motions uniquely defined by the interresidue network connectivity and reveal coupled movements beyond the reach of atomic simulations. Interestingly, the four mutated residues I248, V249, L458, and L459 together with their nearby amino acids F154/F155 reside at the global hinge site of the transporter as determined by GNM (38) (Fig. 5*G*). This mechanical role is consistent with the potential impact of these residues on the structural transitions along the transport cycle. We further note that the other hinge site residues predicted by the GNM here, *i.e.*, D79, Y104, F320, N353, and D421, play critical roles in the transport machinery, coordinating the binding of sodium, chloride, and substrate [reviewed in (14)] (Fig. 5*H*).

Overall, molecular modeling suggests that the TM4-9 mutations alter DAT structure and dynamics in favor of the OF*o* state, either by preventing the progression of the transport from OF to an occluded state or facilitating the transition of the IF state back to the OF state, such that the net effect of the mutations is to increase the population of OF conformers. Cocaine is known to bind and stabilize DAT in the OF state. The mutations would therefore be expected to render the transporter TM4-9 more prone to cocaine binding which will be examined next.

### The quadruple mutant TM4-9 exhibits increased cocaine-binding and altered dopamine-transport properties

**Figure 6:**
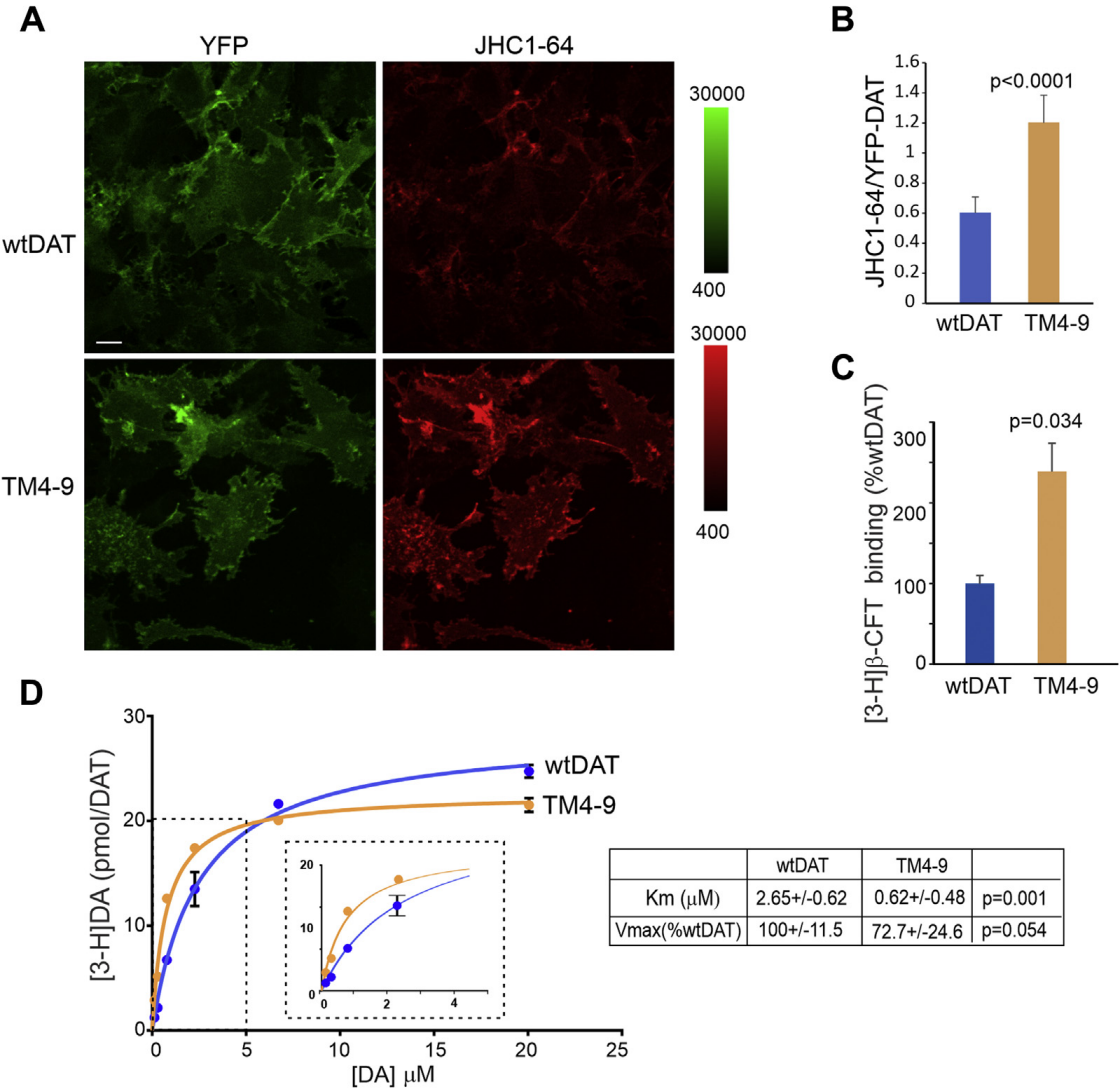
**TM4-9 mutant displays increased cocaine binding and altered kinetics of the substrate transport**. *A*, PAE/YFP-HA-DAT (*wtDAT*) and PAE/TM4-9 cells were incubated with 100 nM JHC 1-64 in F12 supplemented with 10 mM Hepes (pH7.4) for 15 min at room temperature. 3-D images were acquired from fixed cells through 488 nm (YFP) and 561 nm (JHC 1–64) channels. Maximum intensity projections of representative 3D images are shown. Scale bar, 10 μm. *B*, the ratio of JHC 1-64 and YFP fluorescence intensities was calculated from experiments shown in (*A*) as described in “Experimental procedures”. Mean values (±SD; n = 10) are presented. The *p* values were calculated for TM4-9 versus wtDAT using two-tail, unpaired *t* test. *C*, PAE/YFP-HA-DAT (*wtDAT*) and PAE/TM4-9 cells were incubated with 100 nM [3-H]β-CFT at 20 °C for 15 min. Mean values (±SD; n = 3) are presented. The *p* values were calculated for TM4-9 versus wtDAT using two-tail, paired *t* test. *D*, PAE/YFP-DAT (*wtDAT*) and PAE/TM4-9 cells were incubated with 50 nM [3-H]DA and 0.2 to 20 μM cold DA at 20 °C for 10 min. An example of the DA uptake concentration dependence is shown. The amount of bound [3-H]DA was normalized by the amount of YFP-HA-DAT (in arbitrary units) determined by Western blotting with the GFP antibody. Each data point is a mean of triplicate wells. Statistical deviations are not shown if they are smaller than the size of the symbol. Inset shows a detailed view of the part of the graph depicting differences in the DA uptake between WT and mutant DAT at low DA concentrations. Mean values of Km and Vmax were determined from 5 independent experiments. Vmax values are expressed as percent of the Vmax value calculated for WT YFP-HA-DAT in each experiment. The *p* values for TM4-9 versus wtDAT were calculated using two-tail, paired *t* test. AL, AIM-100–like; DAT, dopamine transporter; PAE, porcine aortic endothelial; TM, tansmembrane; YFP-HA-DAT, YFP- and HA-epitope tagged DAT.

To examine the cocaine-binding properties of the TM4-9 mutant, we used a fluorescent cocaine derivative JHC 1-64 because it allows for analyzing binding at subcellular and cellular localization levels. The binding assays were carried out at 20 °C to minimize DAT trafficking during the assay as described previously (25). JHC 1-64 (100 nM) was highly co-localized with the YFP and both accumulated in filopodia and at cell edges, in both WT YFP-HA-DAT and TM4-9 expressing cells. Surprisingly, fluorescence intensity measurements of the ratio of JHC 1-64 and YFP on a pixel-by-pixel basis revealed that JHC 1-64 bound 2-fold more efficiently to the TM4-9 mutant than to the WT YFP-HA-DAT (Fig. 6, *A* and *B*). Similar results were obtained using a radiolabeled cocaine analog, [3-H](2) − 2-β-carbo-methoxy-3-β-(4-fluorophenyl)tropane ([3-H]β-CFT, WIN 35,428) at 100 nM (Fig. 6*C*). These observations confirm the hypothesis derived from MD simulations that the TM4-9 mutant prefers a conformation (OF) predisposed to bind cocaine and that the observed similar mechanisms of action of cocaine and TM4-9 mutations on DAT oligomerization and internalization reflect the shared structural and dynamic alterations induced by either cocaine-binding or quadruple mutations.

The enhanced cocaine-binding properties of TM4-9 prompted us to measure the kinetic parameters of [3-H]DA uptake by this mutant. The mean value of Vmax of the DA uptake by the TM4-9 mutant was found to be slightly smaller than that measured for WT YFP-HA-DAT in five experiments, although the difference was not statistically significant (Fig. 6*D*). By contrast, K_m_ was significantly (∼4-times) smaller for the TM4-9 mutant than WT YFP-HA-DAT (Fig. 6*D*). Therefore, TM4-9 mutant displayed faster substrate transport rate at low micromolar concentrations of DA than WT YFP-HA-DAT, consistent with its structural predisposition to bind cocaine (Fig. 6, *A* and *B*) and facilitated IF-to-OF transition (Fig. 5).

### TM4-9 trimer cannot accommodate AIM-100 or ALs binding in the IF state: WT DAT does

**Figure 7:**
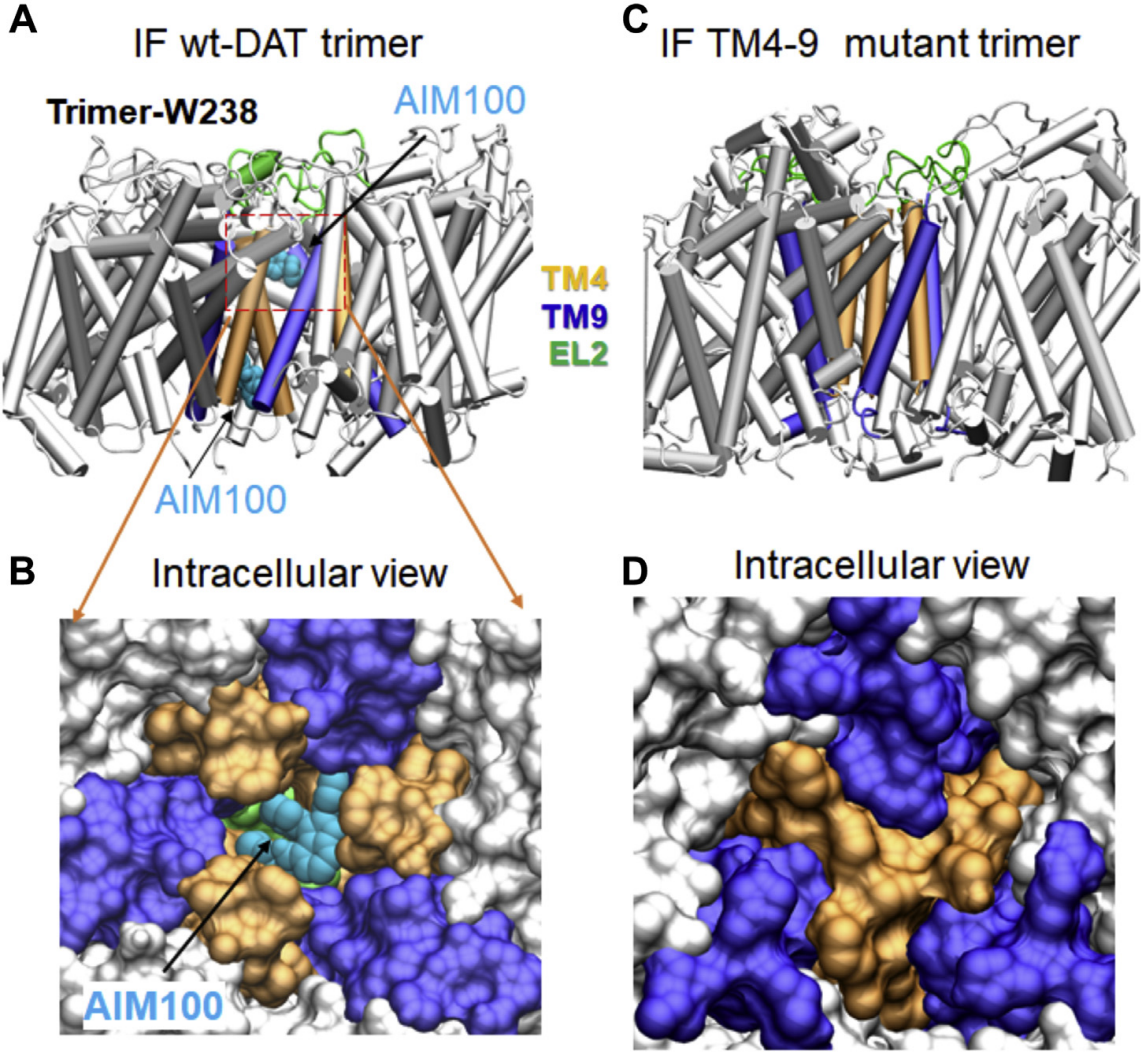
**Dense packing of interfacial residues prevents the binding of AIM-100 to the trimeric interface in the IF TM4-9 mutant**. *A* and *B*, IF WT human DAT trimer. AIM-100 binds to the trimeric cavity in the IF trimer of WT DAT. For more details see (26). *Panel A* shows the side view of the trimer, with two AIM-100 binding sites within the trimeric cavity, and the *panel B* displays the corresponding view from the IC side in space-filling vDW format, showing the AIM-100 bound to the trimeric interface. TM4, TM9, and EL2 loop are colored *orange, violet,* and *green*, respectively. *C* and *D*, side and IC views of IF TM4-9 mutant trimer. The tight packing of the TM helices 4 and 9 obstruct the central cavity that would otherwise bind AIM-100. DAT, dopamine transporter; IC, intracellular; IF, inward-facing; TM, transmembrane; YFP-HA-DAT, YFP- and HA-epitope tagged DAT.

AIM-100 at high concentrations inhibits substrate transport by, and antagonist (*e.g.*, cocaine) binding to, DAT and SERT, thus suggesting its direct binding to the transporters (25, 27). Given the structural and dynamic differences between DAT and TM4-9 mutant, manifested by different effects of AIM-100/ALs on the WT and mutant transporter, we further explored if their AIM-100/AL-binding properties differed. To this end, we simulated the docking of AIM-100/AL onto the WT DAT and TM4-9 mutant monomers in both OF and IF states. AIM-100 bound to S1 or S2 sites within the EC vestibule of in the OF state of both WT DAT and TM4-9 mutant monomers (Fig. S7) but did not show a strong propensity to bind the IF monomers. We further examined the binding of AIM100/ALs to the WT DAT trimer (26) and modeled the TM4-9 mutant trimer. Our previous work showed that AIM-100 tightly binds at the trimeric interface of DAT in the IF state of the “W238” trimer (26). The dense packing at the trimer interface of TM4-9 mutant, on the other hand, prevents AIM-100 binding in the IF state (Fig. 7).

This analysis indicates highly stable, and therefore, SDS-resistant trimers of DAT form upon binding of AIM-100 or AL to the trimeric cavity of WT DAT in the IF state. In contrast, TM4-9 cannot form such highly stable trimers. While both mutant and WT DAT in the OF state can bind those compounds, the difference in their oligomerization and endocytosis properties appears to originate from their distinctive ability to bind these compounds to IF trimers.

### AL effects on DAT oligomerization and endocytosis do not require the N-terminal segment of DAT and an outward-facing conformation of the transporter

**Figure 8:**
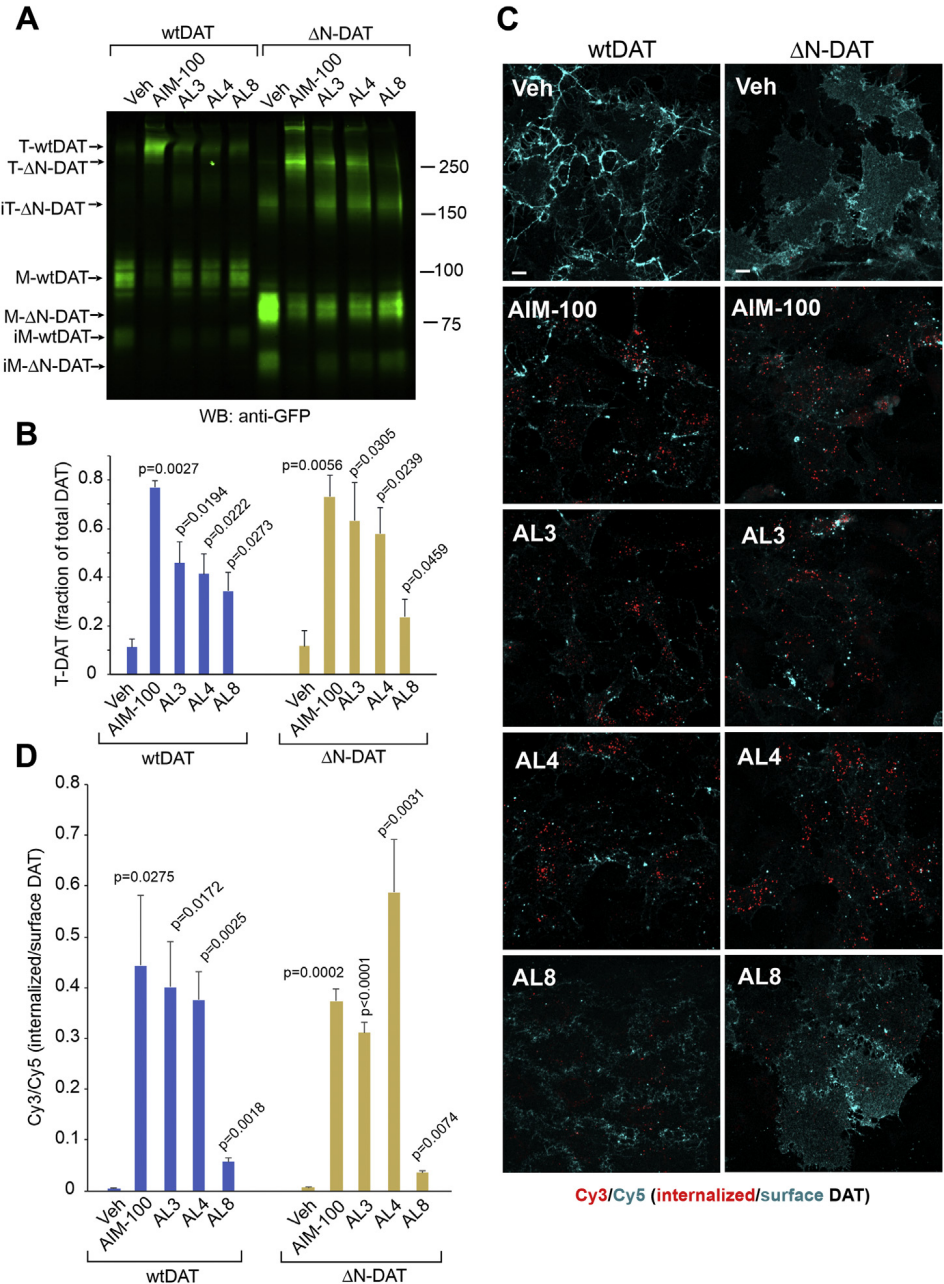
**DAT mutant lacking amino-terminal residues 1-65 is oligomerized and endocytosed upon treatment with ALs.***A*, PAE/YFP-HA-DAT and PAE/YFP-HA-ΔN-DAT cells were incubated with vehicle, AIM-100, AL3, AL4, or AL8 (all 20 μM) for 2 h at 37 °C, and lysates were resolved by SDS-PAGE and immunoblotting with the GFP antibody. Representative experiment is shown. *B*, quantification of the fraction of trimeric species (*T-DAT*) relative to the total YFP-HA-DAT or YFP-HA-ΔN-DAT immunoreactivity in experiments exemplified in (*A*). Bar graph represents mean values of the mature T-DAT fraction of total mature YFP-HA-DAT or YFP-HA-ΔN-DAT (±S.D.; n = 3–4). Differences between the T fractions in YFP-HA-DAT and YFP-HA-ΔN-DAT treated with the same compound were not significant (*p* > 0.05). The *p* values for WT and mutant DATs were calculated for “AIM-100/ALs” versus corresponding “Veh” using multiple comparisons, paired, one-way ANOVA. *C*, PAE/YFP-HA-DAT and PAE/YFP-HA-ΔN-DAT cells were incubated with HA11 for 30 min at 37 °C and then incubated with vehicle (DMSO), AIM-100, AL3, AL4, or AL8 (all 20 μM) for 2 h at 37 °C. After fixation, cells were stained with secondary Cy5-conjugated anti-mouse antibodies (*surface HA-DAT*), permeabilized with Triton X-100, and stained with secondary Cy3-conjugated anti-mouse (*internalized HA-DAT*). 3D images were acquired through 488 (YFP, not shown), 561 (Cy3, *red*), and 640 nm (Cy5, *cyan*) channels. Maximum intensity projections of 3D images are presented. Scale bars, 10 μm. *D*, Cy3/Cy5 ratios were calculated in experiments exemplified in (*C*). Results are presented as mean values of the ratio (±SD). Differences in the ratio values between “YFP-HA-DAT” (*wtDAT*) and YFP-HA-ΔN-DAT (*ΔN-DAT*) treated with the same compound were not statistically significant (*p* > 0.05). The *p* values for WT and mutant DATs were calculated for “AIM-100/ALs” versus corresponding “Veh” using multiple comparisons, paired, one-way ANOVA. AL, AIM-100–like; DAT, dopamine transporter; DMSO, dimethyl sulfoxide; *im-T-DAT*, immature trimers; *im-M-DAT*, immature monomers; *M-DAT*, monomers; PAE, porcine aortic endothelial; *T-DAT*, trimers; YFP-HA-DAT, YFP- and HA-epitope tagged DAT.

The N-terminal segment of this family of transporters plays an important role in maintaining the IC vestibule in a closed form via a network of salt bridges (*e.g.*, R60-D436) or tight interactions (*e.g.*,W63-Y335) (12, 39). Disruption of these interactions impairs IC gate closure, and thereby impedes the progression of the transport cycle to the OF state. Due to this type of destabilization of the OF state, the YFP-HA-ΔN-DAT mutant (that lacks the N-terminal residues 1–65) is unable to transport DA (40). Accordingly, no detectable binding of JHC 1-64 to YFP-HA-ΔN-DAT was observed (Fig. S8). However, the YFP-HA-ΔN-DAT mutant exhibits a strong increase in a 250 kDa species in the presence of AIM-100 or ALs (Fig. 8, *A* and *B*) despite the destabilization of its OF state. This indicates that the OF state is not necessary for AIM-100/AL-induced oligomerization. Elevated levels of monomeric (∼60 kDa) and trimeric (∼180 kDa) immature YFP-HA-ΔN-DAT (Fig. 8*A*) are because of a slightly impaired exit of YFP-HA-ΔN-DAT from the endoplasmic reticulum.

**Figure 9:**
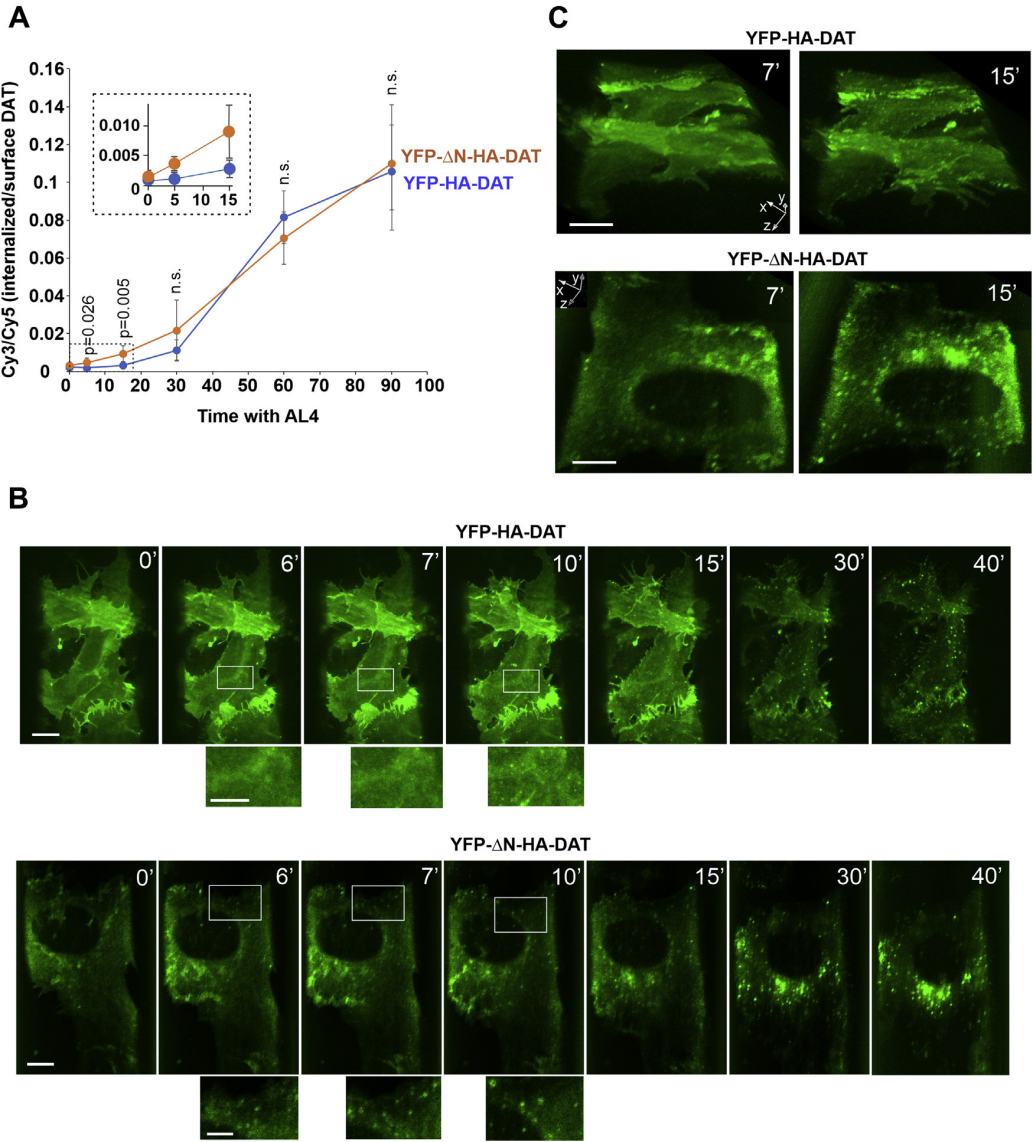
**Initial rate of AL-induced endocytosis of YFP-HA-ΔN-DAT is higher than that of wild-type YFP-HA-DAT**. *A*, PAE/YFP-HA-DAT (WT13) and PAE/YFP-HA-ΔN-DAT cells were incubated with HA11 for 1 h at 37 °C and then incubated with 20 μM AL4 for 0 to 90 min. After fixation, the cells were immunolabeled as in Figure 8*C*. Mean values of the Cy3/Cy5 ratio (±SD, n = 5) were calculated as in Figure 8*D* and plotted against time. The *p* values are shown for YFP-HA-DAT compared with YFP-HA-ΔN-DAT (difference is significant at 5- and 15-min points). Differences were not significant at other time points (n.s., *p* > 0.05). Inset shows part of the graph indicated by the rectangle for better demonstration of different initial endocytosis rates of WT YFP-HA-DAT and YFP-HA-ΔN-DAT. The experiment is representative of three independent experiments. *B* and *C*, Time-lapse 3D imaging of PAE/YFP-HA-DAT (WT13) and PAE/YFP-HA-ΔN-DAT cells incubated with AL4 (20 μM) at 37 °C was performed using the LLS system. Image acquisition intervals were 15 s. Maximum intensity projections (*B*) or “volume” 3D-view images (*C*) of selected 3D images from time-lapse sequences are presented. Scale bars are 10 μm. Insets below images in (*B*) are high-magnification images of the areas indicated by white rectangles. Scale bars, 5 μm. See corresponding Videos S1 and S2 in Supplemental Information. The experiment is representative of multiple time-lapse imaging experiments where similar kinetics of endocytosis were observed in cells treated with AIM-100, AL3, and AL4, whereas AL8 had a much weaker impact on the endocytic process in PAE/YFP-HA-DAT (WT13 and WT8) and PAE/YFP-HA-ΔN-DAT cells. AL, AIM-100–like; DAT, dopamine transporter; LLS, lattice light sheet; PAE, porcine aortic endothelial; YFP-HA-DAT, YFP- and HA-epitope tagged DAT.

In parallel with ALs-increased oligomerization, AL3 and AL4, and to a lesser extent AL8, promoted YFP-HA-ΔN-DAT endocytosis (Fig. 8, *C* and *D*). At the time of maximal accumulation in endosomes, *e.g.,* 2-h incubation with compounds, the WT and mutant transporters exhibited comparable values of the internalized/surface HA11 ratio (Fig. 8*D*). However, a faster accumulation of YFP-HA-ΔN-DAT in endosomes as compared with that of WT YFP-HA-DAT was observed during first 15 min of AL-stimulated endocytosis in time-course experiments, indicative of a higher initial rate of AL-induced endocytosis of YFP-HA-ΔN-DAT (Fig. 9*A*).

To further corroborate this observation at a higher resolution in living cells, the endocytosis dynamics of WT YFP-HA-DAT (WT13) and YFP-HA-ΔN-DAT (similar expression levels) were monitored by time-lapse imaging with a lattice light sheet (LLS) microscopy system. LLS microscopy allows for continuous, extended live-cell three-dimensional (3D) imaging with minimal phototoxicity and photobleaching. In YFP-HA-ΔN-DAT expressing cells, the appearance of YFP puncta was observed after 5 to 7 min of treatment with AL4 whereas at least 10 min was necessary to detect diffraction-limited YFP clusters/vesicles in WT YFP-HA-DAT expressing cells (Fig. 9, *B* and *C*; also see Videos S1 and S2). The number of vesicles that commonly displayed the mobility characteristics of endosomes and gradually accumulated in the juxtanuclear area was higher in cells expressing YFP-HA-ΔN-DAT than in WT YFP-HA-DAT expressing cells at the 15-min timepoint, but the accumulation of both WT and mutant DATs in endosomes was equally massive after 30 to 35 min of AL4 treatment.

In summary, the data in Figures 8 and 9 demonstrate that the removal of the entire N-terminal segment of 65 amino acids and resulting disruption of the OF state of DAT did not thwart the effects of AIM-100 and ALs on DAT oligomerization. On the contrary, the predominant IF state of YFP-HA-ΔN-DAT even appears to accelerate the effects of AL3 and AL4 on DAT endocytosis.

## Discussion

In this study, we identified small molecules structurally similar to AIM-100, termed ALs, which stabilize DAT oligomers and induce DAT endocytosis. Studies of the mechanisms of these AL effects led us to the generation of the TM4-9 mutant, which we believe is the first example of a DAT mutant with both an increased capacity of cocaine binding and a faster substrate transport kinetics at nonsaturating DA concentrations. Comparative analysis of the effects of ALs on DAT and its mutants led us to propose a mechanistic link between DAT conformational state, oligomerization, endocytosis, and activity.

Examination of AL structures and chemical properties suggests that their activity on DAT requires two phenyl and another hydrophobic group attached to a large scaffold such as pyrimidine or triazine, with phenyl groups positioned at ∼75^o^ angle to each other. Lack of the third hydrophobic group eliminates the activity toward DAT (AL1 and AL9), even though their solubilities are similar to those of effective compounds (Figs. 1 and S1). Known DAT ligands GBR12935 and modafinil have two phenyl groups but are ineffective in promoting DAT oligomerization and endocytosis [Fig. 1 and (25)], presumably because of the different angle (>90^o^) between two phenyls and/or lack of the third hydrophobic sidechain. Charged compounds were not effective either, suggesting that their effects involve hydrophobic interactions and/or crossing the lipid bilayer. Importantly, the demonstration that AL3, AL4, and AL8, that are not ACK1 inhibitors, induce oligomerization and clathrin-independent endocytosis of DAT confirms the conclusions from our previous experiments using siRNA and another ACK1 inhibitor KRC0008 (25) that these AIM-100 effects are not because of inhibition of ACK1. However, it is possible that constitutive clathrin-mediated endocytosis is sensitive to ACK1 inhibition, which contributes to AIM-100 effects on DAT trafficking (27).

**Figure 10:**
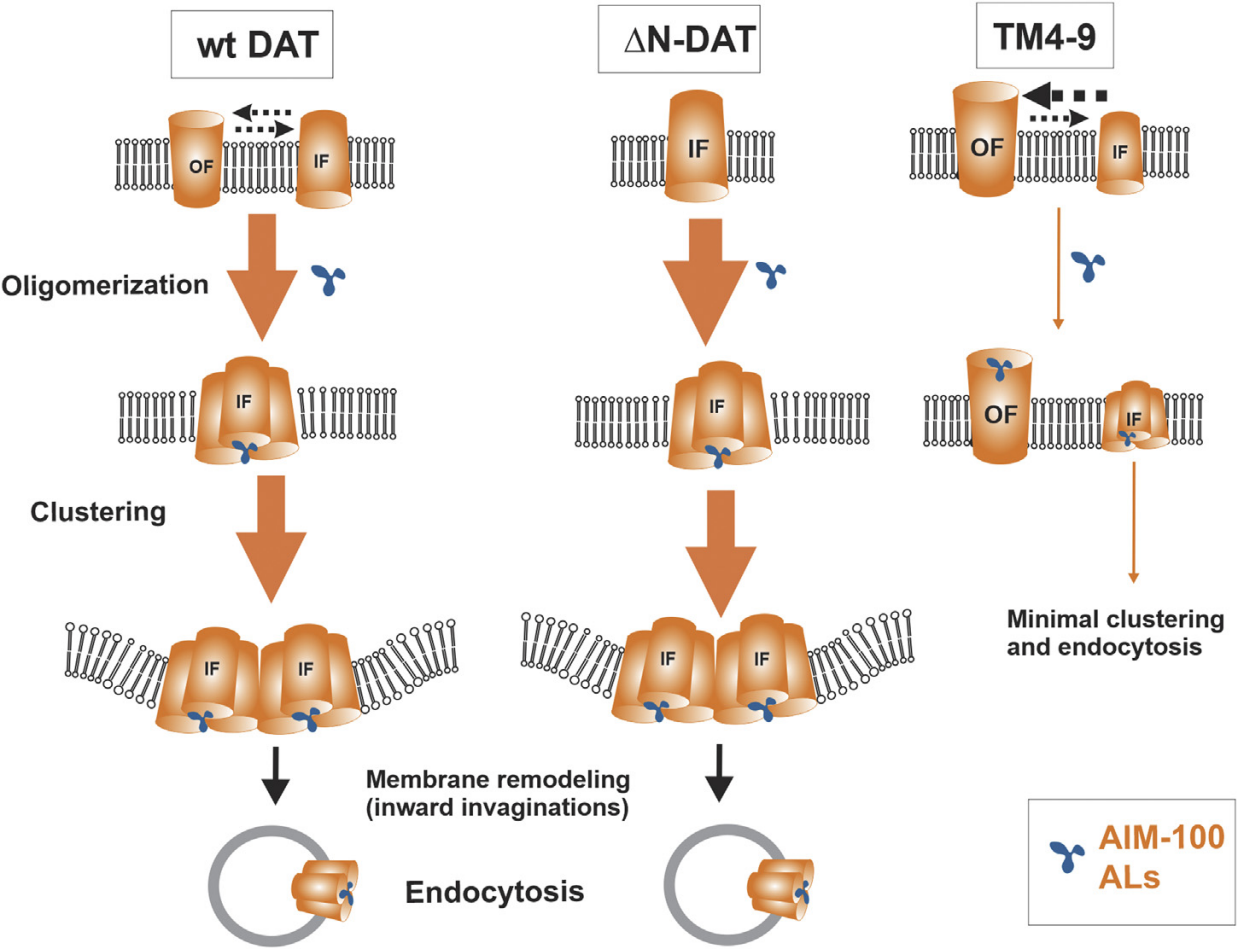
**Hypothetical model of the effects of AIM-100 and ALs on DAT.** AIM-100 and ALs stabilize trimers (oligomers), promote formation of high-order oligomers, and nanoclusters of a fraction of WT DAT in the IF conformation and ΔN-DAT, that is predominantly in the IF state. Clustering of concave-shaped DATs leads to membrane remodeling and stabilizing inward invaginations of the membrane, followed up by vesicle scission (endocytosis). The same ALs and AIM-100 are less efficient in inducing oligomerization of DAT in the OF conformation. For example, the capacity of AIM-100 and ALs to promote oligomerization of TM4-9 is diminished because of the increased probability of the OF state of this mutant. AIM-100 and ALs, are, however, still capable of binding at or near the substrate binding sites of the TM4-9 mutant in its OF state. Widths of arrows roughly reflect process probabilities. AL, AIM-100–like; DAT, dopamine transporter; IF, inward-facing; OF, outward-facing; TM, transmembrane.

In Figure 10, we propose a hypothetical model of how ALs stabilize DAT oligomers and induce endocytosis. The model suggests that AIM-100 and ALs preferably stabilize DAT trimer in its IF state, thus increasing the fraction of these trimers and promoting high-order oligomerization, nanoclustering, and subsequent endocytosis of DAT in the IF state of the DAT molecule.

This model is supported by multiple observations. First, AIM-100 and AL effects on DAT are inhibited by cocaine ( (25) and Fig. 3). This inhibitory effect of cocaine could be because of two possibilities: (i) cocaine and ALs compete for binding to DAT or (ii) cocaine stabilizes the OF fold (41). AIM-100 at high concentrations inhibited the cocaine analog binding to DAT as well as the substrate transport by DAT and SERT (25, 27), which is consistent with its binding to the S1/S2 sites (Fig. 5*S*). However, the EC vestibule and the S1/S2 sites are not accessible in ΔN-DAT while AIM-100 and ALs induce its oligomerization and endocytosis, suggesting that the actions of AIM-100 and ALs do not require binding to S1/S2. Besides, cocaine could not inhibit AIM-100–induced oligomerization and endocytosis in another DAT mutant, W63A, whose EC vestibule was inaccessible (25). Therefore, a more plausible explanation is that the OF state, that can be stabilized by cocaine, is an unfavorable DAT conformation for the induction of oligomerization and endocytosis by AIM-100 and ALs. Interestingly, cocaine and its analogs strongly inhibit cysteine cross-linking of constitutive DAT trimers, tetramers, and higher oligomers (16). The striking similarity of such cocaine effect and its effect on AIM-100/AL-induced oligomerization observed here is indicative of shared mechanisms of DAT oligomerization observed in two different experimental systems.

Second, the TM4-9 mutant displays an increased cocaine analog binding (Fig. 6), explained by the shift in its equilibrium conformation toward the OF state that is predisposed to bind cocaine (Fig. 5). The predominance of the OF state, on the other hand, significantly inhibited the actions of AIM-100 and ALs, again suggesting that the OF state does not lend itself to an actionable binding pose of these compounds.

Finally, the notion that the IF state aids AL-induced DAT oligomerization and endocytosis is supported by the observations that AIM-100 and AL3/4 are fully effective in inducing oligomerization of DAT mutants that are predicted to predominantly exist in the IF state, such as ΔN-DAT (Figs. 8 and 9) and W63A (25), and even more potent in inducing the endocytosis of ΔN-DAT than the endocytosis of WT DAT (Fig. 9). An explanation for an augmented rate of ΔN-DAT endocytosis could be the concave shape of this mutant as it is predominantly in the IF state, whereas a large fraction of WT DAT is in the OF state and convex shape (illustrated in Fig. 10). Concave shape molecules and their oligomers/nanoclusters tend to accumulate in positively invaginated membranes and stabilize these invaginations, which in combination with protein crowding may promote further invagination of the membrane and vesicle scission (Fig. 10). Conversely, convex-shaped OF DATs tend to accumulate in negatively curved membranes, such as filopodia, and even promote the filopodia formation at high DAT concentrations (41). A growing number of examples of membrane remodeling and endocytosis events promoted by clustered wedge-shape TM proteins, molecular crowding, and membrane phase separation have been reported (42, 43, 44, 45, 46, 47, 48, 49). Our live-cell imaging of AL-induced DAT endocytosis using LLS microscopy demonstrated formation of DAT-containing endocytic vesicles but did not detect any tubular endocytic intermediate (Fig. 9; Videos S1 and S2). This observation rules out the contribution of clathrin-independent pathways involving heterogeneous tubular endocytic intermediates, such as CLIC/GEEC, ARF6- and flotillin-dependent endocytosis, in AL-induced DAT endocytosis. Instead, it implicates endocytic mechanisms that involve vesicular intermediates, such as those mediated by membrane protein crowding [reviewed in (50)].

In summary, the inhibitory effects of cocaine as well as TM4-9 mutations on AIM-100/AL-induced oligomerization and the strong effects of AIM-100/ALs on ΔN-DAT and W63A mutants provide solid support for the model of oligomerization and oligomerization-dependent endocytosis of DAT upon stabilization of its 10.13039/100001179IF state by AIM-100 and ALs (Fig. 10). In accord with this model, our molecular simulations show that AIM-100 can bind and stabilize trimers of the WT DAT in the IF state but not TM4-9 mutant whose trimeric interface is too tightly packed to accommodate AIM-100 or ALs binding (Fig. 7).

Chemical cross-linking of cysteines has been shown to trap dimeric, trimeric, tetrameric, and higher oligomeric DAT species suggesting that these species may exist simultaneously (15, 16). AIM-100/ALs stabilize SDS-resistant trimers of YFP-HA-DAT (∼95–100 kDa), YFP-ΔN-DAT (∼85 kDa) and HA-DAT (∼65 kDa) detected by Western blotting as ∼270 to 300 kDa, 250 kDa, and 200 kDa species, respectively, all with the molecular mass 3-fold larger than that of a monomer. These trimers apparently represent only a small fraction of the plasma membrane DAT in untreated cells based on Western blotting detection and cross-linking with BS^3^ and are highly specific to DAT. By contrast, single-molecule microscopy analysis showed that about 35% of the plasma membrane DAT is constitutively dimeric (24). We did not observe SDS-resistant or BS^3^-linked dimer species of WT DAT detectable by Western blotting, and our modeling predicts AIM-100/AL stabilization of DAT trimers by strong hydrophobic interactions, which may explain SDS-resistance of trimers (Fig. 7). Furthermore, DAT dimers were detected by single-molecule imaging because of their lateral mobility in the plasma membrane (24). By contrast, AIM-100 oligomers were mostly immobile (25) and therefore would not be detected by the method used in (24). Certainly, NSS family members may assemble in various dimeric or trimeric forms (51), and some interfacial contacts may be shared between them. Our analyses of AIM-100/AL-stabilized trimers suggest that TM4 and TM9 play an important role in mediating DAT oligomerization in the IF state. Consistent with this behavior is the observation that cross-linking of C243 (TM4) (trimers and tetramers) but not of C306 (TM6) (dimers) was inhibited by cocaine (16). Moreover, recent molecular modeling pointed to the role of TM4 and TM9 in the assembly of symmetric DAT dimers (52). The latter study emphasized that TM2 [proposed to be involved in DAT dimerization in (17)] and TM6 do not significantly contribute to dimerization and oligomerization; whereas multiple interactions between the scaffold domains of DAT can be involved in the dimer or oligomer assembly. Such redundancy is consistent with our mutational analysis, which showed that disruption of a single interaction, *e.g.,* those involving I248 or L459, was not sufficient to fully inhibit AIM-100/AL-induced oligomerization. Likewise, mutations of leucines in TM9, predicted to be part of the leucine heptad repeat, did not interfere with AIM-100 effects on DAT (26).

Concomitant attenuation of the oligomerization response to AIM-100 and ALs (Fig. 4) and altered substrate-transport kinetics (lower Km) of the TM4-9 mutant transporter (Fig. 6) suggests functional and mechanistic links between the regulation of DAT oligomerization and its activity. Although the four mutated residues I248, V249, L458, and L459 do not participate in the substrate- or cocaine-binding sites, they reside in a central hinge region predicted by the GNM (Fig. 5) that mediates the cooperative OF↔IF transitions during the transport cycle (51). As shown in MD simulations (Fig. 5), the quadruple mutation indeed alters the preferred conformation and dynamics in favor of the OF state, and faster substrate transport kinetics could be attributed to the increased probability of external gate opening. In fact, the mechanistic interpretation of conventional Michaelis-Menten parameters in a transporter system is complicated. K_m_ is not solely associated with the substrate binding affinity; instead, it is also determined by the conformational kinetics along the transport cycle, especially the return rate (IF→OF) to reuptake-ready state (53). Thus, the faster IF-to-OF transition in the TM4-9 mutant may lead to faster transport at nonsaturating substrate concentrations.

To conclude, our overall findings suggest that I248-V249 of TM4 and L458-L459 of TM9 can be part of a “hub” for the allosteric regulation of DAT conformation and function. This regulation is highly specific to DAT, making the TM4-9 hub an attractive new target in the design of therapeutic compounds that modulate DAT activity. On the other hand, xenobiotics with the structural similarity to ALs and processes leading to transporter misfolding, such as proteotoxic shock and oxidative damage (for example, by methamphetamine) (54, 55, 56), or a combination of thereof, may cause an increased oligomerization of DAT in its dysfunctional IF state. The coupling of the IF conformation and oligomerization of DAT to its oligomerization-driven endocytosis raises a possibility that such endocytosis may be a part of a peripheral quality-control mechanism eliminating dysfunctional and defective transporters from the plasma membrane.

## Experimental procedures

### Antibodies and chemicals

Antibodies were purchased from the following sources: rat monoclonal antibody against the N-terminus of DAT (MAB369) and rabbit polyclonal antibody to phospho-ACK1 (Tyr284) from EMD Millipore; mouse monoclonal antibody to hemagglutinin epitope HA11 (16B12) from BioLegend; mouse monoclonal antibody to ACK1 from Santa Cruz; mouse monoclonal antibody to GFP (JL-8) from Clontech, rabbit polyclonal antibody to clathrin heavy chain from Abcam. Mouse monoclonal antibody to EEA.1 was from BD Biosciences. Secondary donkey anti-mouse, anti-rat, and anti-rabbit AffiniPure antibodies conjugated with Alexa488, Cy5, and Cy3 from Jackson Immuno Research; IRDye-800 and IRDye-680-conjugated goat anti-mouse, anti-mouse IgG1, anti-mouse IgG2b, anti-rat, and anti-rabbit antibodies were purchased from LI-COR Biosciences. Paraformaldehyde was from Electron Microscopy Sciences. Tissue culture reagents were purchased from Gibco, Thermo Fisher Scientific. AIM-100 was purchased from Tocris Bioscience; phorbol 12-myristate 13-acetate, Triton X-100, protease inhibitors, and most other chemicals were purchased from Millipore Sigma. Precision Plus protein standards were purchased from Bio-Rad. Recombinant human EGF was purchased from BD Biosciences. BS^3^ was from Pierce, Thermo Fisher Scientific. AL1-5 compounds were purchased from ChemBridge, AL 6 to 15 from MilliporeSigma, GBR 12935 from Santa Cruz. AIM-100 and other ALs were stored as 20 to 40 mM stock solutions in dimethyl sulfoxide (DMSO) and diluted in the experimental medium to a final concentration of 10 to 40 μM by vigorous shaking and vortexing or pipetting immediately before incubation with cells. Human recombinant EGF was from Thermo Fisher Scientific.

### DNA constructs and transfections

GFP-fused human NET and GFP-fused human SERT constructs were described previously (25). Site-point mutagenesis was performed on the YFP-HA-DAT template using QuickChange Site-Directed Mutagenesis Kit (Agilent Technologies). DNA constructs were transfected using the Effectine kit (Qiagen).

### Cell culture and transfections

PAE cells were originally obtained from the University of Uppsala (Sweden) (57). PAE cells stably expressing YFP-DAT, CFP-DAT, and YFP-HA-DAT are described in our early studies [for example (18, 30)]. Two clones of wild-type YFP-HA-DAT, WT8 and WT13, expressing high and low levels of the transporter, respectively, were used. PAE cells stably expressing the YFP-ΔN-HA-DAT mutant (clonal pool) were previously described (40). Clonal pools of PAE cells stably expressing GFP-fused human SERT, GFP-fused human NET, or the TM4-9 mutant were generated by G418 selection and cloning. PAE cells stably expressing EGFR-GFP were described previously (33). PAE cells were grown in F12 medium with 10% fetal bovine serum (FBS) without antibiotics. HeLa cells were grown in Dulbecco's modified Eagle's medium containing 10% FBS without antibiotics. HeLa cells were confirmed by genotyping. Cells are regularly checked for *mycoplasma* using Lonza detection kit. The cells were grown on glass coverslips for direct imaging of YFP and immunofluorescence in fixed cells, 35 mm Mat-Tek dishes, or 5-mm coverslips for live-cell imaging, 12-well and 6-well tissue culture plates for biochemical experiments, and 24- or 48-well plates for ligand-binding and uptake experiments.

### Antibody uptake endocytosis assay

The endocytosis assay using HA11 antibody was performed as previously described (25). Briefly, the cells grown on glass coverslips were incubated with 2 μg/ml HA11 in F12 media for 30 min at 37 °C and then in antibody-free F12 medium with various compounds at 37 °C for the indicated times. The cells were washed with ice-cold Hanks balanced salt solution (HBSS) (Thermo Fisher Scientific) and fixed with freshly prepared 4% paraformaldehyde for 15 min at room temperature. The cells were then washed in Dulbecco phosphate buffer saline (DPBS) (Thermo Fisher Scientific) and DPBS containing 0.5% BSA (DPBS/BSA). To maximally occupy surface-DAT bound HA11, cells were incubated for 1 h with excessive concentration (5 μg/ml) of Cy5-conjugated secondary donkey anti-mouse antibody in DPBS/BSA at room temperature. After triple 2-min wash in DPBS/BSA, the cells were permeabilized with 0.1% Triton X-100 in DPBS/BSA for 5 min and then incubated with 0.5 μg/ml of the same secondary antibody conjugated with Cy3 in DPBS/BSA for 45 min at room temperature to label internalized HA11. Unbound antibodies were removed by triple 2-min wash in DPBS/BSA. In some experiments, secondary antibody were used in a reverse order: Cy3-conjugated—to occupy cell-surface HA.11, and Cy5-conjugated—to detect internalized HA11:YFP-HA-DAT complexes. Coverslips were mounted on slides in Mowiol (Calbiochem). All antibody solutions were precleared by centrifugation at 100,000 × *g* for 20 min.

### Fluorescence confocal microscopy

To obtain high resolution 3D images of the cells, a z-stack of confocal images was acquired using a spinning disk confocal imaging system based on a Zeiss Axio Observer Z1 inverted fluorescence microscope (with 63x Plan Apo PH NA 1.4), equipped with a computer-controlled Spherical Aberration Correction unit, Yokogawa CSU-X1 or CSU-W1, Vector photomanipulation module, Photometrics Evolve 16 bit EMCCD camera, environmental chamber, piezo stage controller and lasers (405, 445, 488, 515, 561, and 640 nm), all controlled by SlideBook 6 software (Intelligent Imaging Innovation, Denver, CO). Typically, 15 to 30 serial two-dimensional confocal images were recorded at 200 to 400 nm intervals. All image acquisition settings were identical for all experimental variants in each experiment.

### Antibody uptake assay image analysis

To quantify the relative amount of Cy5 (surface) and Cy3 (internalized) fluorescence in images obtained in the HA11 antibody endocytosis assay, background-subtracted 3D images were segmented using minimal intensities of Cy5 (nonpermeabilized cells staining) and Cy3 (permeabilized cells staining) as low thresholds to obtain Masks #1 and #2, which correspond to the total amount of surface and internalized HA11, respectively. Additionally, a segment mask #3 of Cy3 fluorescence overlapping with Cy5 positive pixels was generated to determine the amount of Cy3-labeled antibodies that bind to surface HA11 because of incomplete occupancy of the surface HA11 with Cy5-labeled secondary antibodies before cell permeabilization. Mask #3 was subtracted from Mask #2 to obtain Mask #4 corresponding to the corrected Cy3 fluorescence (internalized HA11). The integrated voxel intensity (in arbitrary linear units of fluorescence intensity; a.l.u.f.i.) of Masks #1 and #4 were quantitated in each image containing typically 5 to 15 cells, and the ratio of Mask#4 to Mask#1 integrated intensities (Cy3/Cy5 ratio) was calculated to determine the extent of YFP-HA-DAT endocytosis. In experiments where Cy3 and Cy5-conjugated antibodies were used in the reversed order, the Cy5/Cy3 ratio was the measure of YFP-HA-DAT endocytosis.

### Immunofluorescence labeling and co-localization image analysis

PAE cells expressing YFP-HA-DAT were incubated with DMSO (vehicle), AIM-100, or ALs, fixed, permeabilized with 0.1% Triton X-100 in DPBS/BSA for 5 min and then incubated with the EEA.1 antibody followed by secondary Cy3-conjugated antibody in DPBS/BSA at room temperature. Coverslips were mounted on slides in Mowiol. All antibody solutions were precleared by centrifugation at 100,000 × *g* for 20 min, and 15 serial two-dimensional confocal images were recorded at 400 nm z-axes intervals through 488 nm and 561 nm laser channels. All image acquisition settings were identical for all variants in each experiment.

To quantify the relative amount of YFP-HA-DAT localized in early endosomes (EEA.1 positive), a colocalization analysis was performed using Slidebook6. 3D images were deconvolved using a nearest neighbor algorithm of SlideBook6. A segment mask was then generated to select EEA.1 endosomes detected through the 561 nm channel (EEA.1 mask). Another segment mask was generated using automatic segmentation in the 488 nm channel to include total cellular YFP fluorescence (YFP mask). A “Colocalization” mask was then generated to select voxels overlapping in the EEA.1 and YFP masks. The sum fluorescence intensity through the 488 nm channel in the Colocalization mask in each image was divided by the sum intensity of the YFP mask to calculate the fraction of total YFP-HA-DAT located in EEA.1-containing endosomes.

### Lattice light sheet microscopy

Cells grown on 5-mm coverslips were placed in the imaging chamber of a LLS V2 system (Intelligent Imaging Innovation, Inc). Excitation was achieved using 488 diode laser at 2 to 3% AOTF transmittance through an excitation objective (Special Optics 28.6x 0.7 NA 3.74-mm immersion lens) and is detected via a Nikon CFI Apo LWD 25x 1.1 NA water immersion lens with a 2.5x tube lens using Hamamatsu Orca Flash 4.0 V3 sCMOS camera. Live cells were imaged in 3.5 ml of 37 °C-heated FluoroBrite Dulbecco's modified Eagle's medium (Gibco) containing 10% FBS before and after addition of ALs (final concentrations of 20 μM). Typically, 240 3D images (stacks of 101 images at 0.217 μm intervals) were acquired every 15 to 30 s at 37 °C. Time-lapse images were deconvolved using the constrained iterative algorithm of SlideBook6 and presented as time-lapse sequences of maximum intensity projections of 3D images.

### Total internal reflection fluorescence microscopy imaging

Cells in 35 mm Mat-Tek dishes were imaged on a Nikon Eclipse Ti inverted microscope (Nikon) with a 100 x 1.49 NA oil-immersion objective using 488 nm laser line and with 15 s intervals between time frames. All experiments were performed at 37 °C and 5% CO2 in F12 medium. Chemicals were added during continuous image acquisition. Images were collected using Nikon Elements software (version 4.30, Nikon) and a Photometrics95PrimeB camera; at full resolution under these conditions the pixel size with a 1.5x coupler matches Nyquist sampling (70 nm xy exactly).

### FRET imaging and analysis

PAE cells co-expressing YFP-DAT and CFP-DAT (pool of cells expressing various levels of YFP-DAT and CFP-DAT) were described previously (18). The cells grown on 35 mm MatTek dishes were incubated with DMSO (vehicle) or ALs at 37 °C. A method of sensitized FRET measurement that has been described in our studies previously was used (18, 25). Briefly, images were acquired using the spinning disk confocal microscope at room temperature (to avoid artifacts due to the rapid movement of endosomes and filopodia during image acquisition) sequentially through 445 nm (CFP; excitation—445 nm, emission—470 nm), 515 nm (YFP; excitation—515 nm, emission—542 nm) and FRET (excitation at 445 nm; emission at 542 nm) filter channels. All image acquisition parameters were identical in experimental measurements in cells co-expressing CFP-DAT and YFP-DAT and in control measurements of the bleed-through coefficients in cells expressing only CFP-DAT or YFP-DAT which were incubated with DMSO or ALs.

Corrected FRET (FRET^C^) was calculated on a pixel-by-pixel basis using a FRET module of the SlideBook6 software. FRET^C^ images are presented in a pseudocolor mode. FRET^C^ intensity is displayed stretched between the low and high renormalization values, according to a temperature-based lookup table with blue (cold) indicating low values and red (hot) indicating high values. To eliminate the distracting data from regions outside of cells or cells that do not express both CFP and YFP, the CFP channel was used as a saturation channel, and the FRET^C^ images were displayed as CFP intensity-modulated images. In these images, data with CFP values greater than the high threshold of the saturation channel are displayed at full saturation, whereas data values below the low threshold are displayed with no saturation (*i.e.*, black).

FRET^C^ values were also calculated from the mean fluorescence intensities for multiple selected regions of interest (ROI) of the images containing individual ruffles and filopodia (plasma membrane) in vehicle-treated cells, and plasma membrane clusters and endosomes in AL-treated cells as described (25). Normalized sensitized FRET (FRETN) values for individual subcellular structures, regions, and compartments were calculated according to the equation (1): FRETN = FRET^C^/(YFP x CFP), where FRET^C^, CFP, and YFP are the mean background-subtracted intensities of FRET^C^, CFP, and YFP fluorescence in the ROI. Because FRETN displays a nonlinear dependence when donor or acceptor are in a significant molar excess over each other, FRETN values were calculated in ROIs in which the relative stoichiometry of the donor and acceptor was not more than 3. All calculations were performed using SlideBook6.

### Detection of DAT by Western blotting

The cells rinsed with ice-cold HBSS were solubilized in TGH (1%Triton X-100, 10% glycerol, 20 mM HEPES, 50 mM NaCl) lysis buffer supplemented with 1% deoxycholate, and protease (including iodoacetamide and MG132) and phosphatase inhibitors for 30 to 60 min at 4 °C on low speed shaker. Lysates were centrifuged at 16,000 × *g* for 10 min to remove insoluble material. Aliquots of cell lysates were denatured in Laemmli buffer (2%SDS, 5% 2-mercaptoethanol, 10% Glycerol, 62.5 mM Tris HCl, pH6.8) for 5 min at 95 °C, resolved by 7.5% SDS-PAGE, transferred to nitrocellulose (Li-COR), and probed with appropriate primary and secondary antibodies conjugated to far-red fluorescent dyes (IRDye-680 or -800) followed by detection using an Odyssey Li-COR system. Quantifications were performed using Li-COR and ImageJ software. Molecular mass of stained Precision Plus protein standards was verified by Coomassie Blue staining of unlabeled Precision Plus protein standards from Bio-Rad.

### Cell-surface biotinylation

The cell-surface biotinylation was performed as previously described (25) with some modifications. The cells grown in 12-well or 6-well plates were incubated with vehicle or ALs for 2 h at 37 °C, washed with ice-cold HBSS, and incubated with 1 mg/ml Sulfo-NHS-biotin (Thermo Fisher Scientific) for 40 min at 4 °C in HBSS and then quenched with 100 mM glycine-HCl buffer. After rinsing with HBSS, the cells were solubilized in the TGH lysis buffer supplemented with 1% deoxycholate and inhibitors as listed above, also containing 10 mM Tris HCl (pH6.8) for 30 min at 4 °C on low speed shaker. Lysates were centrifuged at 16,000 × *g* for 15 min to remove insoluble material. Aliquots of lysates were taken for input control, and the rest of lysates was incubated with NeutroAvidin-Agarose (Thermo Fisher Scientific) at 4 °C on nutator for 1 h to pull-down biotinylated proteins. The beads were triple-washed in lysis buffer supplemented with 100 mM NaCl (NaCl was omitted in the last wash) for 3 min at 4 °C on nutator. NeutroAvidin precipitates and aliquots of cell lysates were denatured in Laemmli buffer for 5 min at 95 °C, resolved by 7.5% SDS-PAGE, transferred to nitrocellulose (Li-COR), and probed with appropriate primary and secondary antibodies conjugated to far-red fluorescent dyes (IRDye-680 or -800) followed by detection using Odyssey Li-COR system. Quantifications were performed using Li-COR and ImageJ software.

### Chemical cross-linking

The cells grown in 12-well plates were incubated with vehicle or ALs for 1 h at 37 °C, double-washed with Krebs-Ringer HEPES-glucose buffer (KRHG; 120 mM NaCl, 4.7 mM KCl, 2.2 mM CaCl_2_, 1.2 mM Mg SO_4_, 1.2 mM KH_2_PO_4_, 10 mM glucose, 10 mM HEPES, pH 7.4), and incubated with 4 mM BS3 for 15 min at 37 °C in KRHG followed by quenching with 100 mM glycine-HCl buffer. The cells were solubilized, lysates resolved by 7.5% SDS-PAGE and probed by Western blotting as described above in biotinylation experiments.

### DA uptake assays

The cells were grown to confluence in 24- or 48-well culture plates. The cells were rinsed with KRHG. The cells were then incubated with 50 nM [3-H]-DA (PerkinElmer Life Sciences) and either 2 μM or a range of increasing concentrations of unlabeled DA (0.08, 0.24, 0.74, 2.2, 6.6, and 20 μM) in KRHG supplemented with 10 μM pargyline and10 μM ascorbic acid for 10 min at room temperature. The DA uptake was terminated by quickly washing the cells three times with 0.5 ml ice-cold KRHG. Cells were then solubilized in 0.1 N NaOH/1% SDS. Cell-associated [3-H]DA was determined by liquid scintillation counting. Nonspecific [3-H]DA accumulation was determined in the presence of 50 μM cocaine. The amount of cell-associated DA was normalized by the amount of mature species (∼90–100 kDa monomer and ∼270–300 kDa trimer) of WT YFP-HA-DAT or its mutant measured by SDS-PAGE and Western blotting with the GFP antibody in several wells which were processed exactly as the wells used for the DA uptake measurement in the same multiwell plate. K_m_ and V_max_ values for [3-H]DA uptake were calculated using Michaelis-Menton equation by GraphPad software.

### Measurement of β-CFT binding

Binding of [3-H]β−CFT to DAT was measured by incubating cells with 2.5 nM [3-H]β−CFT supplemented with unlabeled β−CFT (97.5 nM) in F12 medium containing 10 mM Hepes (pH7.4). Binding assays were carried out in 48-well plates in the F12 medium for 15 min at 20 °C, conditions minimizing DAT trafficking. Nonspecific binding was determined in the presence of 50 μM cocaine or using parental PAE cells. The lack of detectable YFP-DAT endocytosis during the assay was confirmed in cells grown in parallel MatTek dishes by live-cell imaging and cell-surface biotinylation. β-CFT binding was terminated by three washes with ice-cold F12 medium. Cells were then solubilized in 0.1 N NaOH/1% SDS. Cell-associated [3-H]-β−CFT was measured by liquid scintillation counting. The amount of cell-associated β−CFT was normalized by the amount of mature species (∼90–100 kDa monomer and ∼270–300 kDa trimer) of WT YFP-HA-DAT or its mutant measured by SDS-PAGE and Western blotting with GFP or DAT antibodies in several wells which were processed exactly as the wells used for the β−CFT binding measurement in the same multiwell plate.

### DAT oligomerization analysis in synaptosomes

All experimental procedures were performed in strict accordance with the recommendations in the Guide for the Care and Use of Laboratory Animals of the National Institutes of Health. All the animals were handled according to the approved Institutional Animal Care and Use Committee protocol (#16088832) of the University of Pittsburgh. Striatal synaptosomes were prepared as described (58). Synaptosomes were equilibrated in modified KRHG (140 mM NaCl, 5 mM KCl, 2 mM CaCl_2_, 1 mM MgCl_2_, 5.5 mM HEPES and 10 mM D-glucose, pH 7.4) at 4 °C for 2 h, pelleted by centrifugation at 12,500 × *g* for 20 min at 4 °C, and re-suspended in fresh KRHG (one striatum in 0.9–1.2 ml) by pipetting 10 times with 1 ml pipet tip. Equal aliquots of re-suspended synaptosomes were incubated with vehicle (DMSO) or 20 to 40 μM AIM-100 or ALs for 2 h at 37 °C on nutator, centrifuged at 12,500 × *g* for 20 min at 4 °C, re-suspended in KRH/G and centrifuged again. Pelleted synaptosomes were solubilized in TGH lysis buffer (1%Triton X-100, 10% glycerol, 20 mM HEPES, 50 mM NaCl) supplemented with 1% deoxycholate and protease (including iodoacetamide and MG132) and phosphatase inhibitors for 60 min at 4 °C on nutator. Lysates were centrifuged at 16,000 × *g* for 10 min to remove insoluble material. Equal aliquots of the lysates were denatured by heating in Laemmli buffer for 30 min at 42 °C and processed for SDS-PAGE/Western blot analysis as described above.

### Measurement of JHC 1-64 binding

JHC 1-64 was a kind gift from Dr Amy Newman (National Institutes of Drug Abuse). For JHC 1-64 binding experiments, cells were incubated with 100 nM JHC 1-64 in F12 media (supplemented with 10 mM Hepes) at room temperature (RT) for 15 min and fixed. A z-stack of 15 confocal images at 300 nm z-steps was acquired through 488 nm (YFP) or 561 nm (JHC 1–64) laser channels using a single dichroic. All images were captured using the same exposure time and all other image acquisition parameters.

Images were analyzed using SlideBook6. Background was subtracted in each image. To quantitate JHC 1-64 binding, segmental mask #1 was generated to select YFP-containing voxels in each image and mask #2 to select JHC 1-64 containing voxels. The mask #3 that contained the overlapping fluorescence of both fluorophores was then generated. The mean ratio of JHC 1-64 and YFP fluorescence intensities (in arbitrary linear units of fluorescence intensity) was calculated using mask #3 in each 3D image.

### Molecular dynamics simulations of WT DAT and TM4-9 DAT in the of and if states

The OF*o* TM4-9 DAT was generated based on dopamine-bound OF*o* WT DAT (37), with the substitutions (I248F, V249T, L458F, and L459F) made using CHARMM-GUI (59). Inward-facing open (IF*o*) TM4-9 DAT and WT DAT were constructed based on the most recent cryo-EM structure of hSERT IF*o* conformer (PDB: 6DZZ) (60), following established homology modeling protocols (37). Briefly, for each protein, 100 models were generated using MODELLER (61), and the one with the lowest MODELLER objective function score was selected for further refinement by MD. MD simulation systems were prepared using CHARMM-GUI Membrane Builder module (59). Each protein was embedded into 1-palmitoyl-2-oleoyl-sn-glycero-3-phosphocholine membrane lipids; and TIP3P waters and Na^+^ and Cl^−^ ions corresponding to 0.15 M NaCl solution were added to build a ∼110 × 110 × 118 Å^3^ simulation box. E491 was protonated, and a disulfide bond was added to C180 and C189 (37). For the OF*o* TM4-9 DAT, the positions of one DA, two sodium ions, and one chloride ion were adopted from WT DAT (37). Each simulation system contained ∼131,000 atoms, one transporter, approximately 300 lipid molecules, and 27,000 water molecules. All simulations were performed using NAMD (62) (version NAMD_2.12) following previous protocol for WT OF*o* (59) and IF*o* (63) DATs. For each OF*o* or IF*o* conformer, at least two independent runs of 200 ns were performed. In all runs, the root-mean-square deviation from the original structure reached 2.0 ± 0.4 Å around 50 to 70 ns and remained almost flat during the rest of simulations, indicating that the mutant was structurally stable. Visual molecular dynamics (64) with in-house scripts was used for visualization and trajectory analysis

### Modeling of TM4-9 trimers

We generated *in silico* a series of trimeric models for TM4-9 using the trimer docking module of the software *ClusPro* (65), following the docking protocol we used for modeling WT DAT trimer (26). For the OF trimer, we used as input an OF conformer equilibrated in the presence of DA and two Na^+^ and one Cl^-^ ions; and for the IF state, the conformer reached after equilibration. We generated 30 and 23 clusters of conformations for the respective IF and OF TM4-9 trimers, upon clustering ∼1000 trimeric models generated by ClusPro (65). The clusters were rank-ordered by size and further selected based on structural criteria specific to NSS multimers (26): (1) cylindrically symmetric or pseudo-symmetric organization of the monomers; (2) exposure of the N termini and C termini to the IC region; (3) suitable orientation of the aromatic residues (*i.e.*, Trp) on the EC and IC sides to enable effective anchoring to the membrane.

### Trajectory, global dynamics

Visual molecular dynamics (64) with in-house scripts was used for visualization and trajectory analysis. The global dynamics and hinge sites of DAT were evaluated using the GNM (38) as implemented in *ProDy* (66). In the GNM, a protein is simplified by a network of nodes (Cα of amino acids) connected by the springs. The connectivity of the network is defined the *N × N* Kirchhoff matrix **Γ**, the off-diagonal elements of which are −1 if nodes *i* and *j* are within rc ≈ 7.0 to 7.5 Å for folded proteins and zero otherwise (67). The equilibrium dynamics of the folded protein is described by a set of N-1 normal modes (68). The cross correlations between the fluctuations ΔR_*i*_ and ΔR_*i*_ of residues *i* and *j* are found from (38): <ΔR_*i*_·ΔR_*j*_>=(3*k*_*B*_T/γ)[Γ^−1^]_*ij*_, here, *k*_*B*_ is the Boltzmann constant, *T* is the absolute temperature, γ is the force constant assumed to be uniform for all springs in the network, and [**Γ**^−1^]_*ij*_ is the *ij*^*th*^ element of the inverse of **Γ**. The kth eigenvector (uk) of Γ gives the profile of residue displacements along the mode k. The global hinges are identified from the crossover between the positive and negative elements of the eigenvectors u1 and u2 (69). More details regarding calculation of hinge sites can be found in (69).

### Statistical analysis

Statistical significance (*p* values) were calculated using unpaired or paired two-tailed Student’s *t* test or one-way ANOVA with Tukey’s or Fisher SLD multiple comparison tests (GraphPad and Excel). Specifics are indicated in Figure legends.

## Data availability

All the data described in the manuscript are contained within the manuscript.

## Supporting information

This article contains supporting information.

## Conflict of interest

The authors declare no conflicts of interest in regards to this manuscript.
