# Supplementary material for: Direct coupling of oligomerization and oligomerization-driven endocytosis of the dopamine transporter to its conformational mechanics and activity
Source: J Biol Chem. 2021 Feb 18;296:100430. doi: 10.1016/j.jbc.2021.100430 (PMC8010718; doi:10.1016/j.jbc.2021.100430)
Supplement: Figures S1–S8 and video legends [file mmc1.pdf]

Supplemental Information – Sorkina et al. Direct coupling of oligomerization and oligomerization-driven endocytosis of the dopamine transporter to its conformational mechanics and activity

Figure S1. Experimentally tested AIM-100-like (AL) compounds and other DAT ligands.

Action	Compound	PubChem CID	Structures[a] 2D 3D	Volume[b] (Å^3)	Apolar desolvation energy (kcal/mol)[c]	Polar desolvation energy (kcal/mol)[d]
Effective	AIM-100	11501591	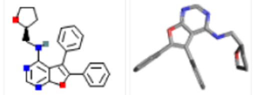	309.9	10	-9.82
	AL3	2142801	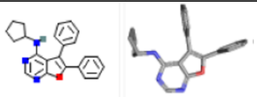	301.5	-0.96	-8.42
	AL4	1037836	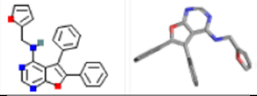	301.8	-0.32	-9.74
Partially effective	AL8	70588	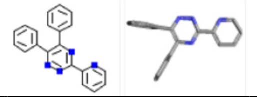	260.3	9.34	-12.7
	AL2	1068329	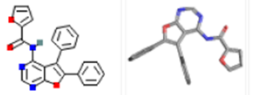	304.0	0.38	-13.6
	AL5	1596636	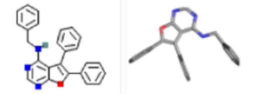	318.3	-0.84	-8.88
ineffective	AL1	1583275	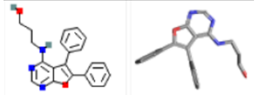	287.3	7.22	-10.3
	AL6	30976	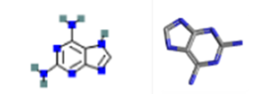	115.1	1.37	-10.0
	AL7	3830	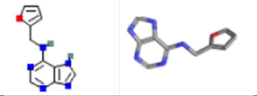	169.2	5.13	-10.7
	AL9	252499	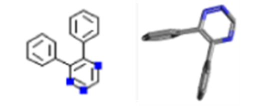	196.9	1.25	-7.65
	AL10	10614	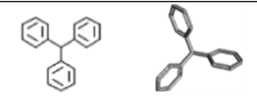	220.8	11.81	-3.25
	AL11	6406861	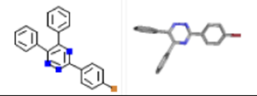	276.6	11.94	-7.32
	AL12	23662871	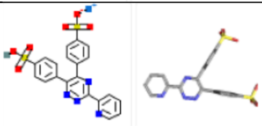			

	AL13	10232	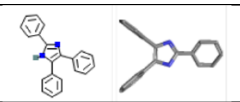	247.4	11.8	-8.79
	AL14	69588	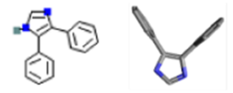	183.9	8.91	-8.48
	AL15	679479	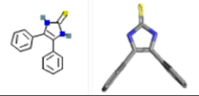	202.1	8.99	-11.26
	AL16<br>GBR 12935	11957553	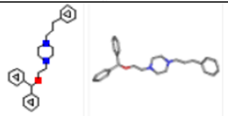			
Other drugs/ligands	Cocaine	446220	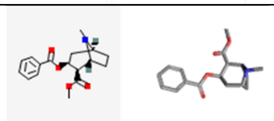	248.1	3.63	-32.1
	Modafinil	4236	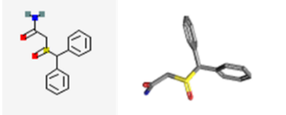	223.4	4.58	-19.4
	dopamine	681	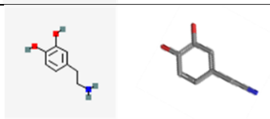	128.4	-1.62	-50.2
	Amphetamine	3007	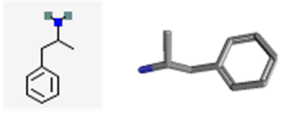	129.6	4.01	-39.1

[a]*2D and 3D structures taken from PubChem*
[b]*molecular volumes calculated through* *http://www.scfbio-iitd.res.in/software/drugdesign/VolumeCalculator.jsp*
[c]*apolar desolvation energy are taken from ZINC* [d]*polar desolvation energy taken from ZINC*
Effective, partially effective, ineffective compounds, and DAT ligands are shaded in orange, green and blue and white.

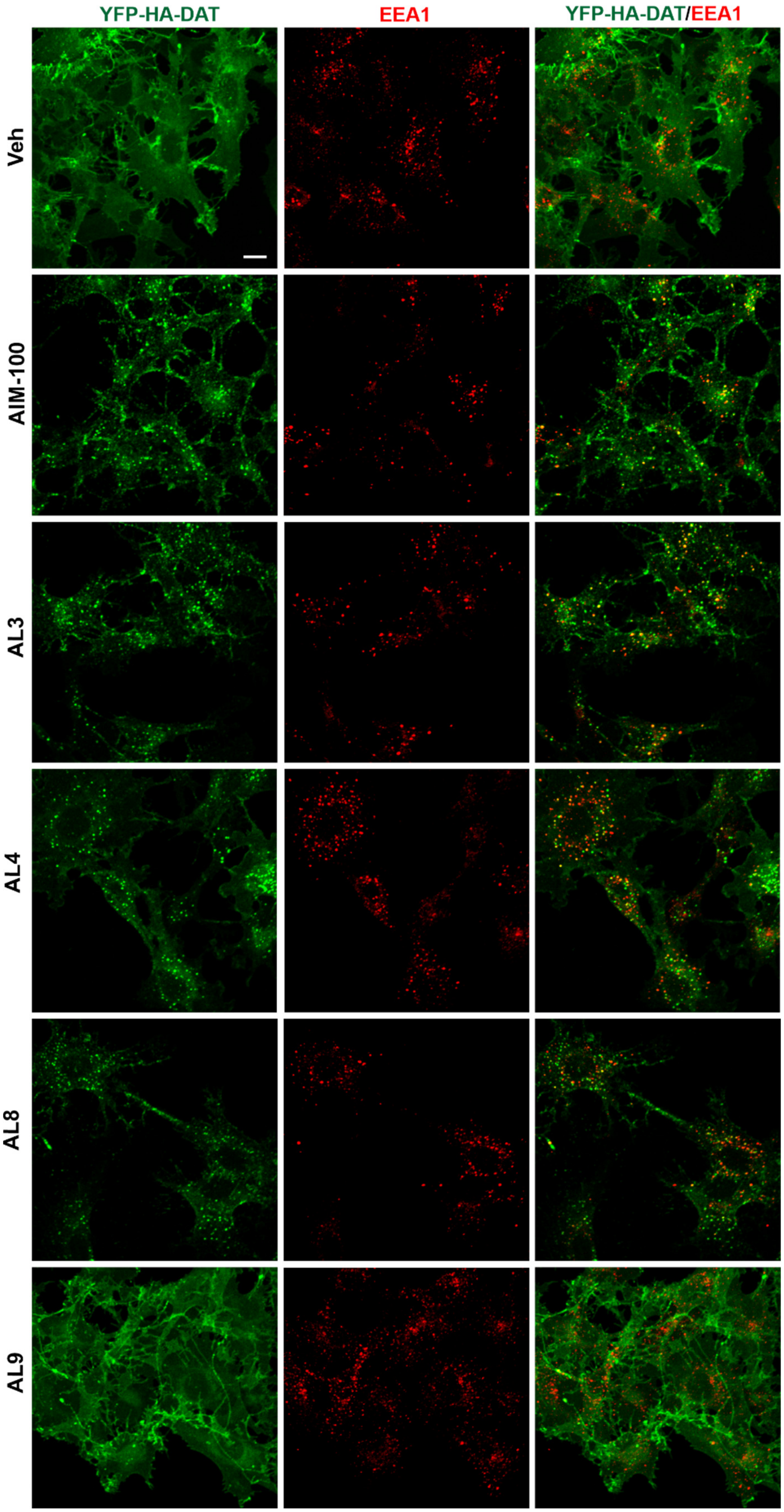
YFP-HA-DAT
EEA1
YFP-HA-DAT/EEA1
Veh
AIM-100
AL3
AL4
AL8
AL9

Figure S2. Effect of AIM-100 and ALs on YFP-HA-DAT internalization into early endosomes.

PAE/YFP-HA-DAT cells were incubated with vehicle (DMSO), AIM-100, AL3, AL4, AL8 or AL9 (all 30 μM) for 2 hrs at 37ºC, fixed and immunolabeled with EEA.1 antibody. 3D images were acquired through the 488-nm (YFP, *green*) and 561-nm (EEA1, *red*) channels. Maximum intensity projections of representative 3D images are presented. Scale bar, 10 μm.

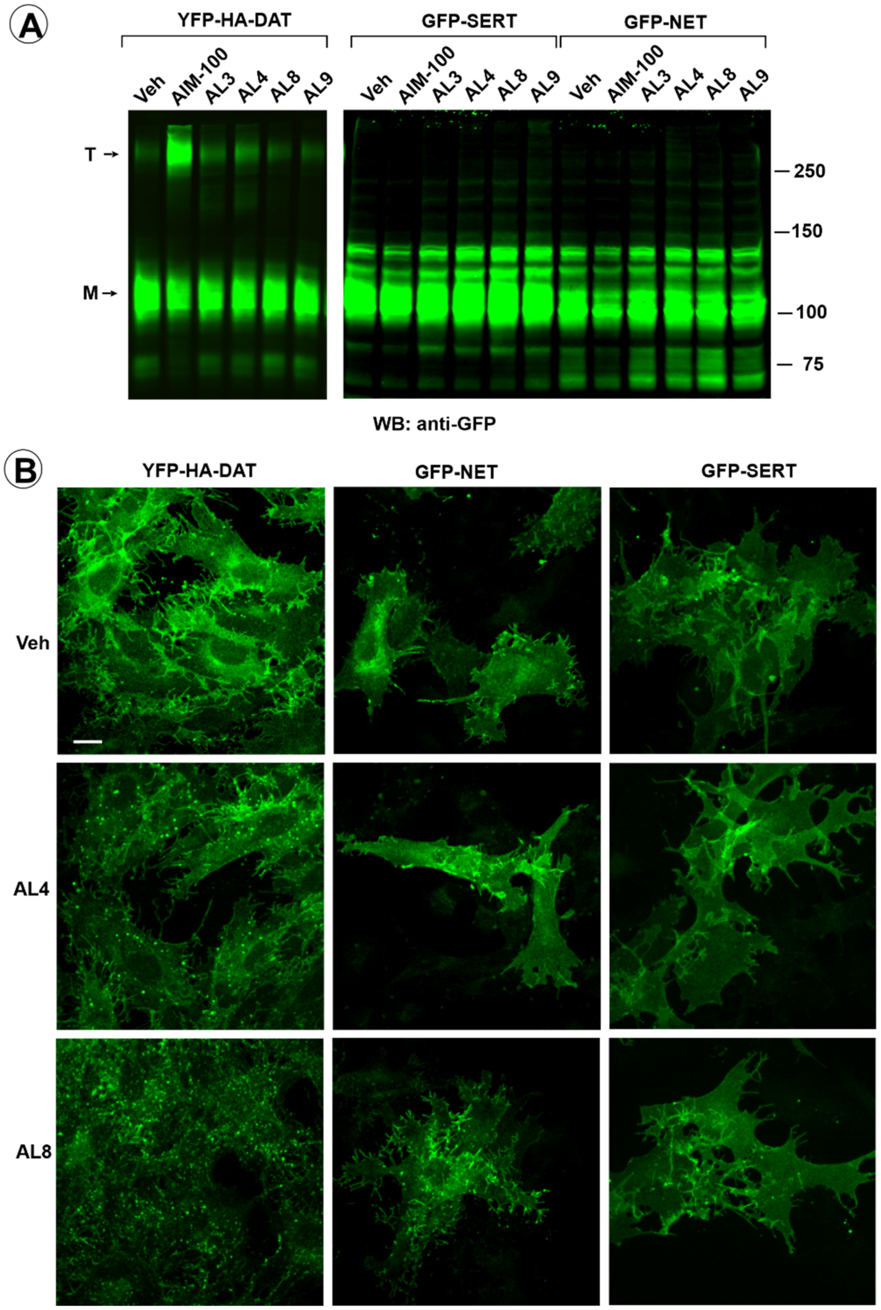

Figure S3. ALs do not enhance oligomerization of NET and SERT or cause their accumulation in endosomes. (A) PAE cells stably expressing YFP-HA-DAT, GFP-NET or GFP-SERT were incubated with vehicle (*Veh*; DMSO), AIM-100, AL3, AL4, AL8 or AL9 (all 20 μM) at 37ºC for 2 hrs. Lysates were resolved by SDS-PAGE and probed by western blotting with the GFP antibody. Blots of YFP-HA-DAT and GFP-NET/GFP-SERT are from the same gel. *M*, monomers; *T*, trimers. **(B)** PAE cells stably expressing YFP-HA-DAT, GFP-NET or GFP-SERT were incubated with vehicle (DMSO), AL4 or AL8 (all 20 μM) for 2 hrs at 37ºC. 3D images were acquired from fixed cells through the 488-nm (YFP, *green*). Maximal intensity projections of representative 3D images are presented. Scale bar, 10 μm. Data are representative of three independent experiments.

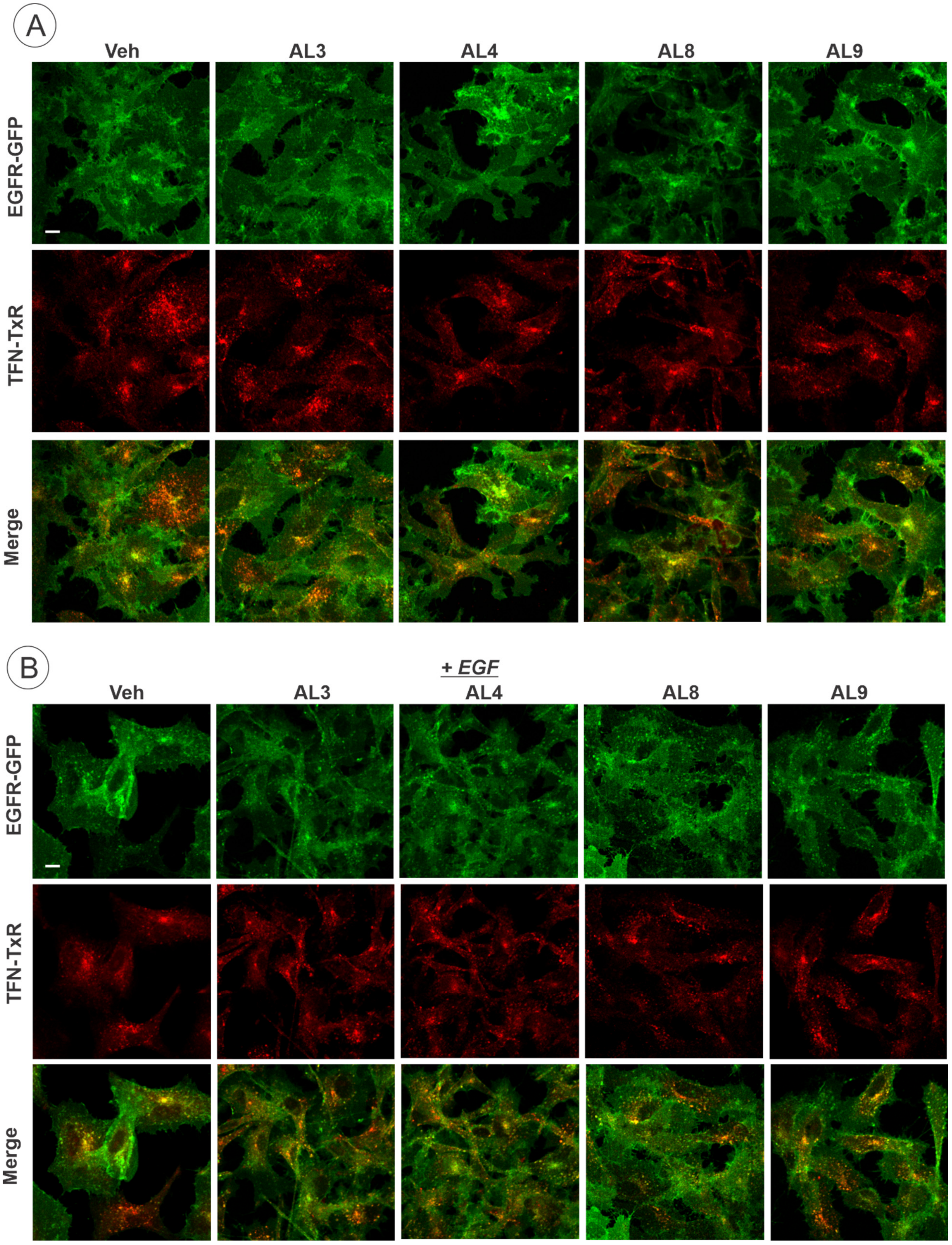
A
Veh
AL3
AL4
AL8
AL9
EGFR-GFP
TFN-TxR
Merge
B
+ EGF
Veh
AL3
AL4
AL8
AL9
EGFR-GFP
TFN-TxR
Merge

Figure S4. ALs do not affect constitutive and EGF-stimulated endocytosis of EGFR-GFP and endocytosis of transferrin.

(**A**) PAE/EGFR-GFP cells were incubated with DMSO (veh), AL3, AL4, AL8 or AL9 (all 40 μM) for 2 hrs at 37ºC, and then incubated with 5 μg/ml TexasRed-conjugated transferrin (TFN-TxR) for 15 min at 37ºC. Cells were fixed and 3D images were acquired through 488 nm (green, GFP) and 561 nm (red, TexasRed) channels. Representative individual confocal sections through the middle of cells are presented. Scale bars, 10 μm.

(**B**) PAE/EGFR-GFP cells were incubated with DMSO (veh), AL3, AL4, AL8 or AL9 (all 40 μM) for 2 hrs at 37ºC, and then incubated with 5 μg/ml Tfn-TxR and 100 ng/ml EGF for 15 min at 37ºC. Cells were fixed and 3D images were acquired through 488 nm (green, GFP) and 561 nm (red, TexasRed) channels. Representative individual confocal sections through the middle of cells are presented. Scale bars, 10 μm.

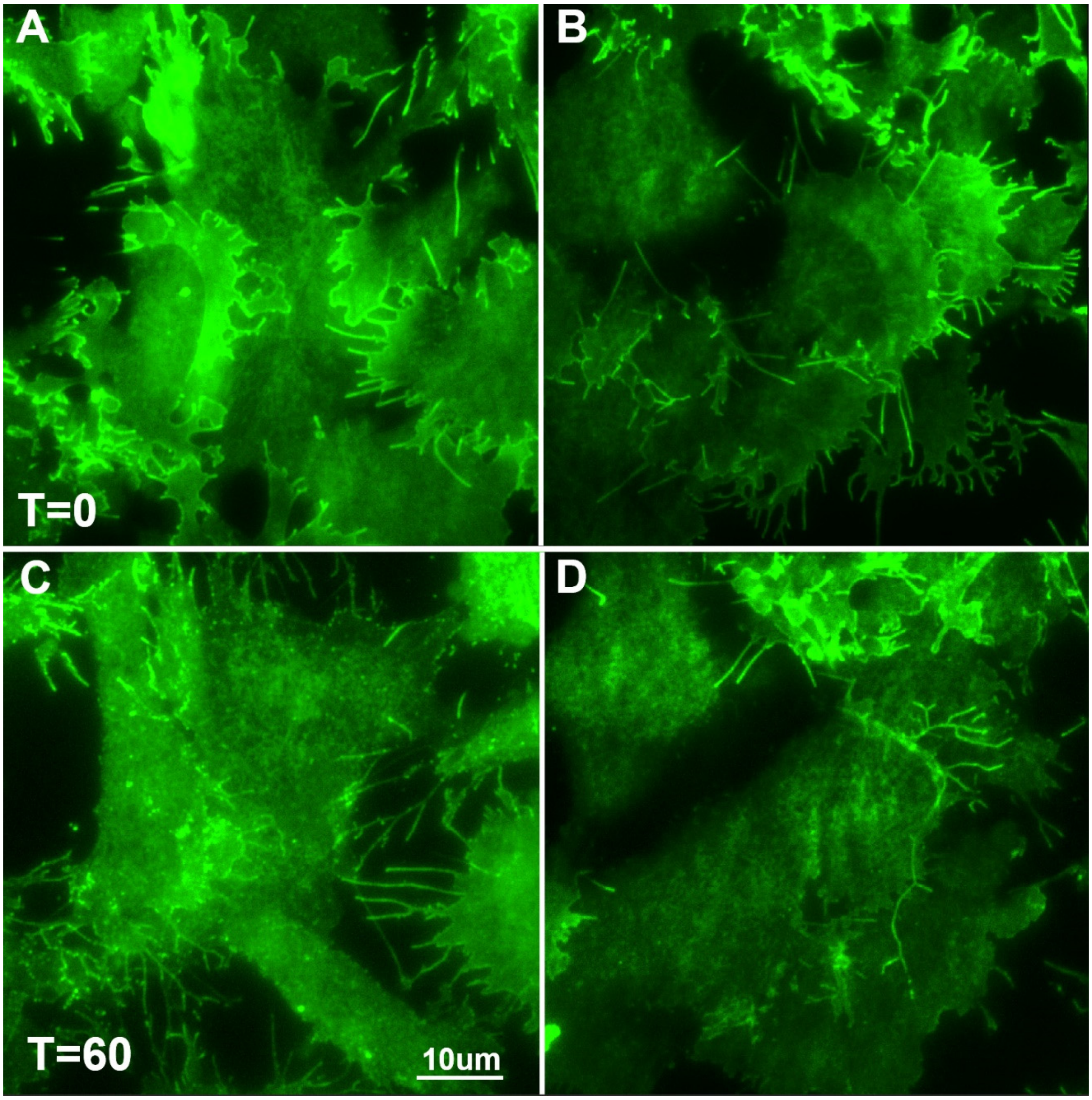

Figure S5. Time-lapse TIR-FM imaging of PAE/YFP-HA-DAT (**A** and **C**) and PAE/TM4-9 (**B** and **D**) cells. Individual time frames before (T=0) and 60 min (T=60) after addition of AIM-100 (20 μM) are shown. Note the reticular tubular fluorescence that represents endoplasmic reticulum situated in close proximity to the bottom-cell plasma membrane. See Video S3.

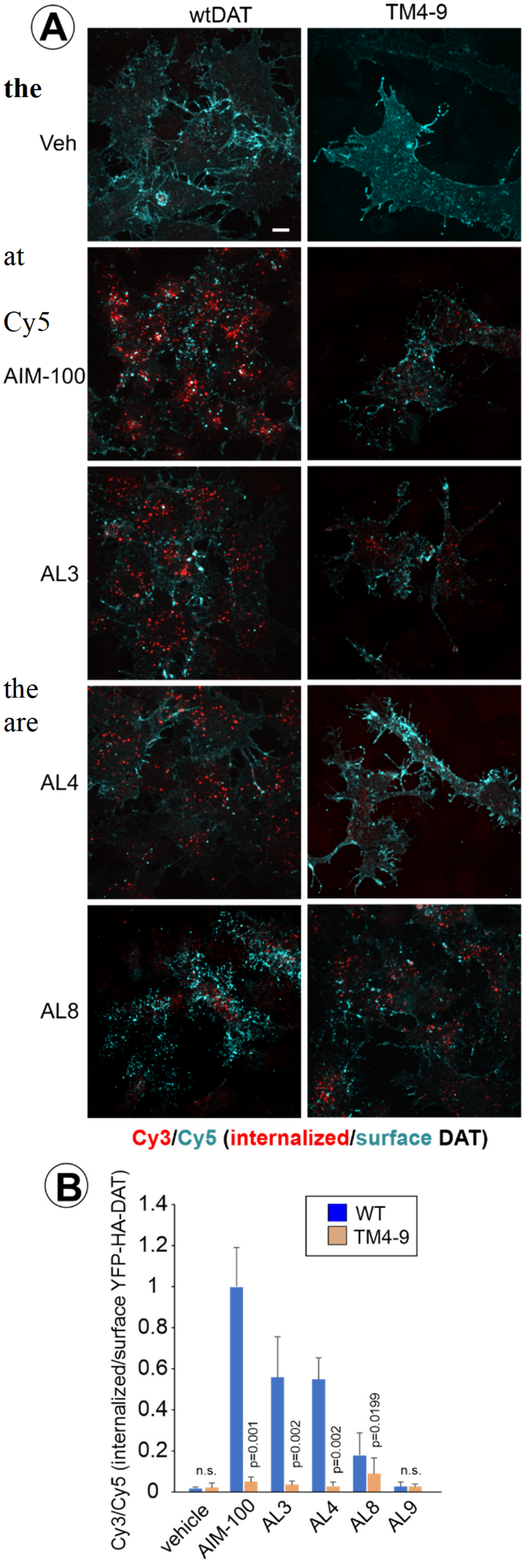

Figure S6. Mutations in TM4 and TM9 inhibit effects of ALs on DAT endocytosis.

(A) PAE/YFP-HA-DAT and PAE/TM4-9 cells were incubated with HA11 for 60 min at 37ºC, and then incubated with vehicle (DMSO), AIM-100, AL3, AL4 or AL8 (all 20 µM) for 2 hrs 37ºC. After fixation, cultures were stained with secondary anti-mouse antibodies conjugated with (*surface HA-DAT*), permeabilized with Triton X-100 and stained with secondary anti-mouse conjugated with Cy3 (*internalized HA-DAT*). 3D images were acquired through 488 (YFP, not shown), 561 (Cy3, *red*) and 640 nm (Cy5, *cyan*) channels. Maximum intensity projections of representative 3D images are shown. Scale bar, 10 µm.

(B) Cy3/Cy5 ratios were calculated in experiments exemplified in *(A)*. Values of the ratio in different experiments are normalized to ratio value in cells treated with AIM-100. Results presented as mean values of the ratio (+/-S.D.; n=9-12). P values for “wt DAT” versus “TM4-9” for each compound treatment were calculated using two-tail, unpaired T-test. *n.s.*, not significant.

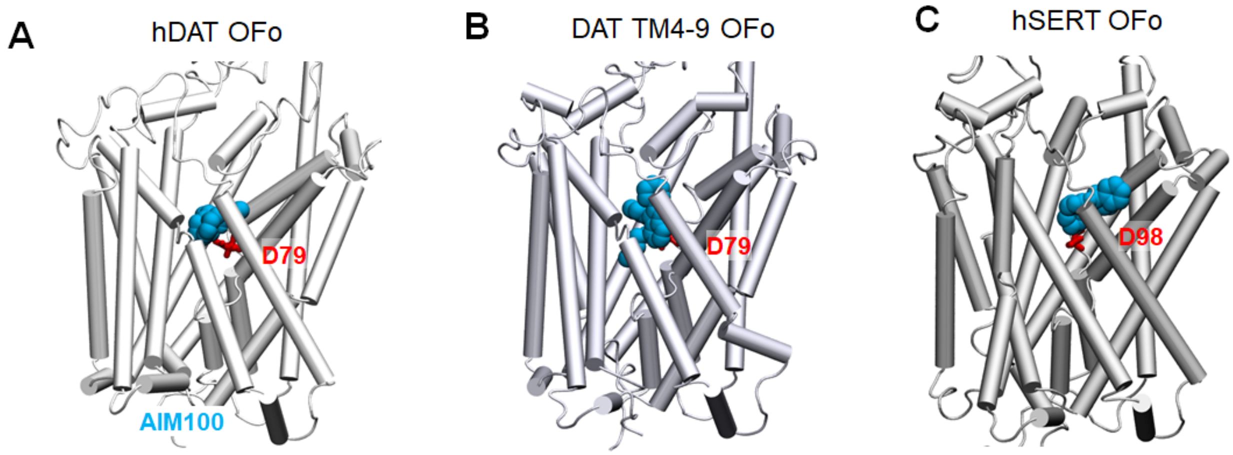

Figure S7. Most probable binding poses of AIM-100 to the outward-facing open conformer of (**A**) human DAT; and (**B**) DAT TM4-9 mutant, in monomeric state. In the OFo conformation, AIM-100 or AL molecules (not shown) are predicted to bind to S1 or S2 sites within the extracellular vestibule. The AIM-100 binding affinity was calculated to be -6.8±0.5 kcal/mol, and -7.1±0.5kcal/mol, for OFo hDAT and TM4-9 mutant, respectively.

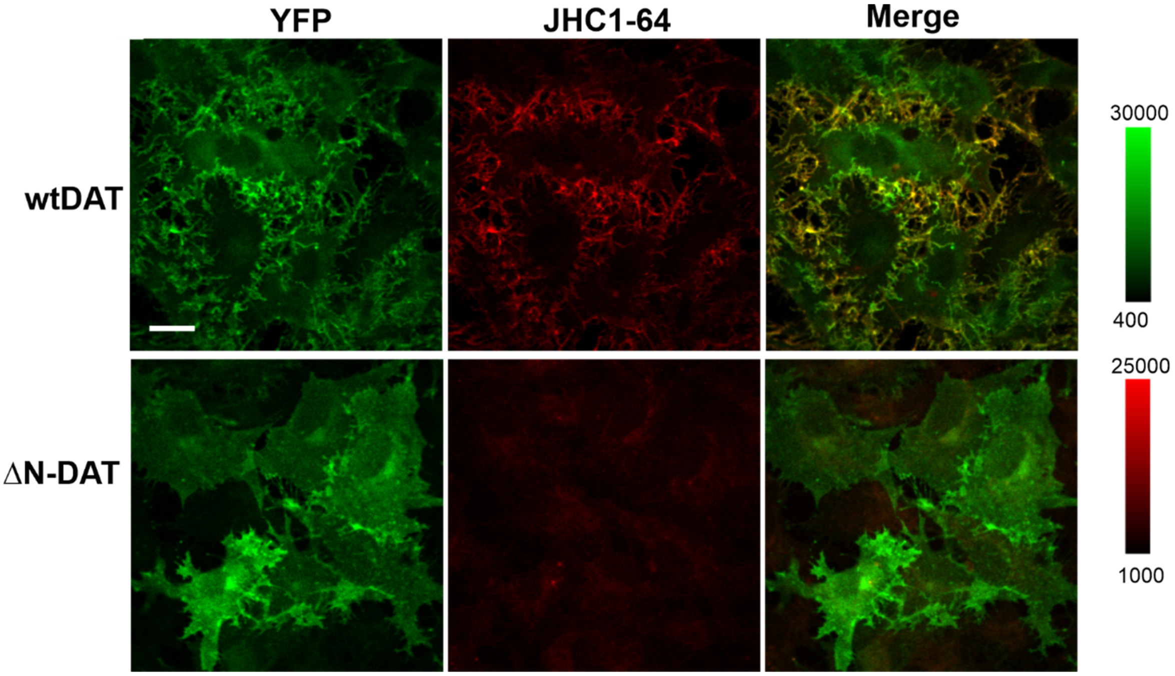

Figure S8. YFP-HA-ΔN-DAT mutant does not bind JHC 1-64.

PAE/YFP-HA-DAT (*wtDAT*) and PAE/YFP-HA-ΔN-DAT cells (*ΔN-DAT*) were incubated with 100 nM JHC 1-64 in F12 supplemented with 10 mM Hepes (pH7.4) for 15 min at room temperature. 3-D images were acquired from fixed cells through 488 nm (YFP) and 561 nm (JHC 1-64) channels. Individual confocal sections of representative 3D images are shown. Scale bar, 10 μm.

Video captions

Video S1. Time-lapse 3D imaging of PAE/YFP-HA-DAT (WT13) cells incubated with AL4 (20 μM) at 37ºC was performed using the LLS system. Image acquisition intervals were 15 sec. Maximum intensity projections of 3D images are presented. Scale bar, 5 μm.

Video S2. Time-lapse 3D imaging of PAE/YFP-HA-ΔN-DAT cells incubated with AL4 (20 μM) at 37ºC was performed using the LLS system. Image acquisition intervals were 15 sec. Maximum intensity projections of 3D images are presented. Scale bar, 5 μm.

Video S3. Time-lapse TIR-FM imaging of PAE/YFP-HA-DAT and PAE/TM4-9 cells was performed at 37ºC before and after addition of AIM-100 (20 μM).
